# Metabolite Profiling and Antioxidant Activities in Seagrass Biomass

**DOI:** 10.3390/md23050193

**Published:** 2025-04-29

**Authors:** Pilar Garcia-Jimenez, Milagros Rico, Diana del Rosario-Santana, Vicent Arbona, Marina Carrasco-Acosta, David Osca

**Affiliations:** 1 Department of Biology, Faculty of Marine Sciences, Instituto Universitario de Investigación en Estudios Ambientales y Recursos Naturales IUNAT, Universidad de Las Palmas de Gran Canaria, 35017 Las Palmas, Spain; diana.delrosario@ulpgc.es (D.d.R.-S.); marina.carrasco@ulpgc.es (M.C.-A.); david.osca@ulpgc.es (D.O.); 2 Department of Chemistry, Instituto Universitario de Oceanografía y Cambio Global-IOCAG, Universidad de Las Palmas de Gran Canaria, 35017 Las Palmas, Spain; milagros.ricosantos@ulpgc.es; 3 Departament de Biologia, Bioquímica i Ciències Naturals, Universitat Jaume I, 12071 Castelló de la Plana, Spain; arbona@uji.es

**Keywords:** antioxidants, *Cymodocea nodosa*, metabolites, *Posidonia oceanica*, ramets, seeds

## Abstract

In this work, metabolite profiling of seeds and antioxidant analysis of fragments of two marine seagrasses, *Posidonia oceanica* and *Cymodocea nodosa,* were carried out to identify metabolite signature involved in seed viability and to evaluate the potential of fragments as a source of bioactive compounds. Using HILIC/QTOF-MS, UHPLC-MS and spectrophotometric analysis, seed metabolites and polyphenols and antioxidant activities, such as those of radical scavenging (RSA), reduction (FRAP, CUPRAC) and complexation (CCA), of rhizome fragments were evaluated. Metabolite comparison between seeds revealed differences across development stages (germinated and non-germinated) and seed types (dormant and non-dormant), providing insights into metabolic activity potentially associated with germination processes and seed viability. Furthermore, polyphenol analysis showed the highest content of caffeic acid in mature leaves (17.00 ± 0.02 μg g^−1^ dw for *P. oceanica* and 98.00 ± 0.03 μg g^−1^ dw for *C. nodosa*). Total phenolic content was correlated with flavonoids and with reduction and complexation activities. The combination of radical scavenging activity and t_1/2_ was higher in *P. oceanica* than *C. nodosa* and also surpassed the commercial synthetic antioxidant BHA. We conclude *P. oceanica* and *C. nodosa* exhibit distinct seed metabolite profiles related to germination and type of seeds, and that fragments are rich in antioxidants, with potential as sustainable sources of bioactive compounds.

## 1. Introduction

Metabolomic approaches have been proposed for the detection and characterisation of compounds involved in crucial plant physiological processes, including seed behaviour during germination [1,2,3,4]. In particular, the identification of specialised metabolites offers insights into potential biomarkers and their associated bioactivities while also revealing how environmental conditions affect plant metabolic dynamics [5,6,7]. Among the main classes of plant metabolites, lipids—including isoprenoids and steroids—serve structural and signalling roles, although several functions remain to be clarified [8]. Phenolic compounds, including flavonoids, lignans and phenolic acids [9,10], are also widely recognised for their physiological significance in plants, particularly in oxidative stress protection [11,12], growth and reproduction [13]. In general, flavonoids are widely distributed in plants and include compounds such as chalcones and (iso) flavones. Lignans are represented by secoisolariciresinol diglucosides, while phenolic acids can be categorised into a general group known as organoheterocyclic compounds, which includes hydroxybenzoic acids, acetophenones, phenylacetic acids and hydroxycinnamic acids.

In contrast to terrestrial plants, seagrasses have been less explored in terms of secondary metabolites. Nonetheless, secondary metabolites, such as zosteric acid and caffeic acid, have been discovered [14,15,16], highlighting the potential of these marine species as valuable biochemical resources. In terms of seed analysis, seagrass seeds can exhibit different behavioural and physiological states, outlining stages of dormancy and non-dormancy. *Posidonia oceanica* seeds are in a non-dormant state and can be transported away from the plant mother and germinate in the meantime [17], whereas *Cymodocea nodosa* seeds are in a dormant state and settle close to the mother plant [18]. This difference enables investigation of the metabolic profiles associated with dormancy and viability as disruption of seed dormancy is followed by seed germination [19,20,21]. Furthermore, rhizome fragments allow the assessment of biochemical resources which could be relevant for potential biotechnological exploitation.

As *P. oceanica*, an endemic species in the Mediterranean Sea, and *C. nodosa*, widely distributed in the Canary Islands (Spain), are protected species and ecosystems, seeds and ramets (i.e., fragments containing rhizome, roots and leaves) washed up on the coast can be exploited due to inconvenience of obtaining a sustainable supply of biomass from meadows for large scale production of such compounds. This biomass can be predictably found throughout the year depending on certain atmospheric and oceanographic conditions [22,23]. Under these expected conditions, biomass washed ashore from seagrass meadows can be an alternative to study target metabolites as well as its contribution to the characterisation of biocompounds and assessment of antioxidant activities. This study focuses on (i) the identification of potential metabolites through HILIC/QTOF-MS in two developmental stages of seeds (germinated and non-germinated) of both *P. oceanica* and *C. nodosa* to unveil potential metabolites associated with germination processes and (ii) the assessment of phenolic compounds and antioxidant capacities of seagrass fragments to be used as a sustainable source of natural antioxidants. These findings contribute to the valorisation of seagrass residues, demonstrating their potential as valuable sources of bioactive compounds.

## 2. Results

### 2.1. Global Analysis of Seed Metabolites

**Figure 1:**
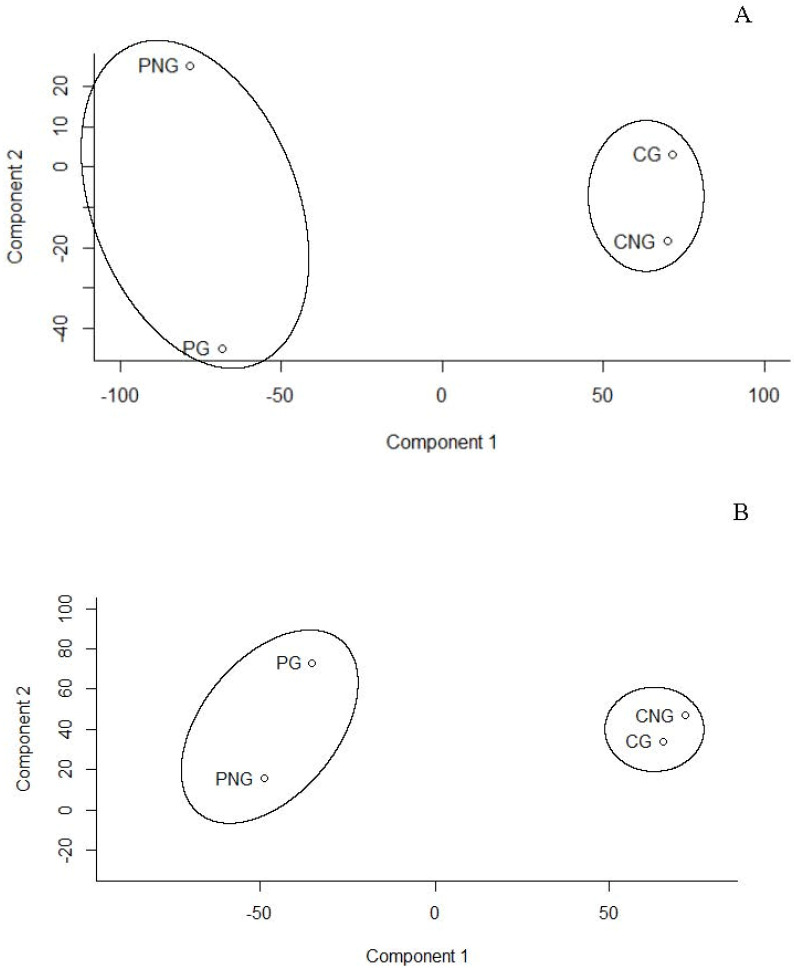
Principal component analysis (PCA) for metabolites of *Posidonia oceanica* (P) and *Cymodocea nodosa* (C) for germinated (G) and non-germinated (NG) seeds according to (**A**) positive and (**B**) negative modes. Points in PCA are the result of analysis performed by compilation of metabolites from negative and positive mode analysis with pooled seeds for each stage. This means non-germinated (PNG) and germinate (PG) seeds of *P. oceanica* and non-germinated (CNG) and germinate (CG) seeds of *C. nodosa.* Note that four points in the PCA are the result of analysis performed by compilation of metabolites from negative and positive mode analysis with pooled seeds for each stage.

Principal Component Analysis (PCA), a multivariate statistical technique, is applied to metabolite datasets to uncover patterns by identifying directions of greatest variance. Data are then transformed into new variables called principal components that summarise variation. To continue, components are ranked according to the amount of variance they capture, helping to reveal metabolic differences between two seed types (*Cymodocea* and *Posidonia*) and their developmental stages (germinated and non-germinated seeds) under both positive and negative ionisation modes. Thus, the results of the PCA reveal that the first two principal components (PC1 and PC2) explain most of the variance in the data: 85% and 10%, respectively, in negative mode and 84% and 12% in positive mode (Figure 1). The analysis shows that all samples representing two different marine plants and two seed development stages are grouped into two clusters corresponding to *C. nodosa* and *P. oceanica* (Figure 1A,B). These findings indicate significant metabolic differences between the two marine plants. Furthermore, differences between germinated and non-germinated seeds of *P. oceanica* were also observed (Figure 1A,B)

### 2.2. Metabolites in Seeds of Seagrasses

**Figure 2:**
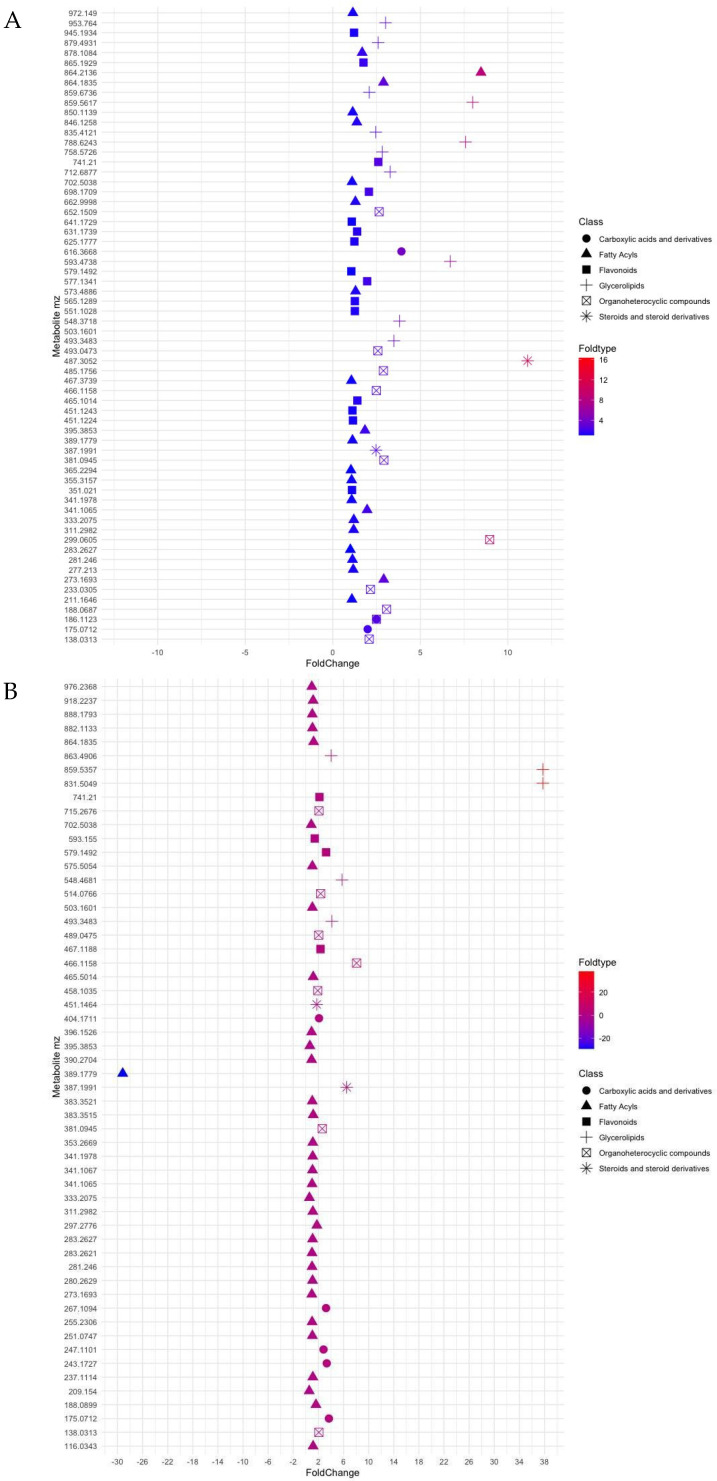
Plot showing log2 fold change values (*x*-axis) for ratios *m*/*z* of metabolites of (**A**) *Posidonia oceanica* and (**B**) *Cymodocea nodosa*, classified according to six groups, namely derivatives from tricarboxylic acid cycle, fatty acyls, flavonoids, glycerolipids, organoheterocyclic compounds and steroids.

Metabolite data using the estimated compound mass [M] (considering all features as [M + H]^+^ or [M − H]^−^) allowed the detection of 1964 peaks of known and unknown metabolites (Tables S1 and S2). Metabolites were mainly classified into phenolic compounds (flavonoids), organoheterocyclic compounds, carboxylic acids and derivatives involved in cellular responses towards tricarboxylic acid cycle (TCA cycle) and lipids. For facilitating analysis, lipids were further subdivided into glycerolipids, fatty acyls and steroids for both seagrasses. Analysis of the filtered data using default ppm of 0–10 ppm mass error and values of germinated/non-germinated ratios for each compound (average value of all ≥1 ratio) resulted in significant changes in the metabolite profiles of both seagrasses and seed stages (Figure 2).

**Figure 3:**
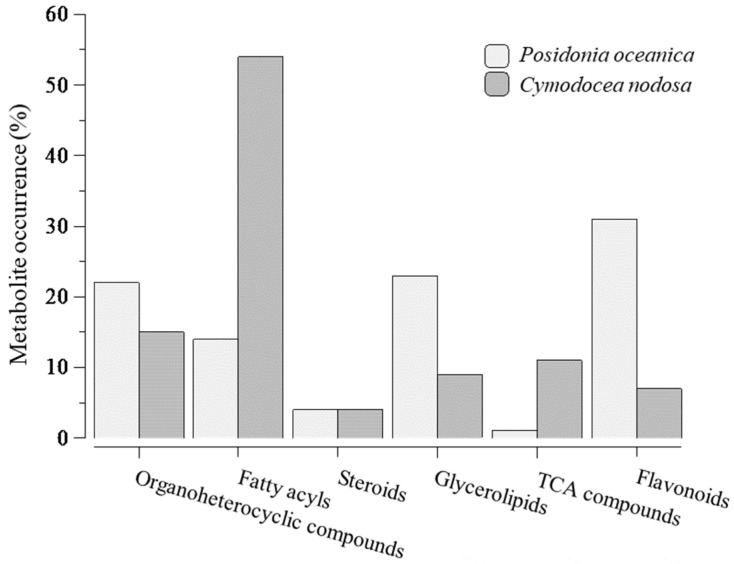
Distribution of metabolites by categories, namely organoheterocyclic compounds, fatty acyls, steroids, glycerolipids, compounds from tricarboxylic acid cycle and flavonoids, after analysis according to ratios over average value of all ≥1-ratios and metabolites selection constrained to a default 0–10 ppm mass error. 100% for *Posidonia oceanica* = 51 metabolites and for *Cymodocea nodosa* = 54 metabolites.

Under constrictions, the metabolite profile of seeds of *P. nodosa* and *C. nodosa* has provided a chemical fingerprint of how metabolites changed with seed maturation, i.e., germinated/non-germinated ratio. Thus, most of the metabolites identified in *P. oceanica* were reported 3- and 4-fold higher in germinated seeds (Figure 2A), whereas non-significative changes were observed between two development stages of seeds of *C. nodosa*, with the exception of some lipid compounds (Figure 2B). Furthermore, the metabolite profile is dominated by the presence of fatty acyls in *C. nodosa* with 54% of the total metabolites, and flavonoids (31%) and glycerolipids (23%) in *P. oceanica* (Figure 3).

Exploration of metabolites of *C. nodosa* through ratio assessment (Table S3) showed that glycerophospholipids (PG) were well-represented in germinated seeds (*m*/*z* 831.5049; ratio 37) of *C. nodosa* and, to a lesser extent, by ceramides, phosphatidylcholines (PC) and phosphatidylinositols (PI) with ratios ranging from 4- (*m*/*z* 863.4906) to 5.7-fold times (*m*/*z* 548.4681). In *P. oceanica*, the presence of glycerophospholipids was also reported abundantly in germinated seeds with ratios ranging between 6.7 (*m*/*z* 593.4738) and 7.99 (*m*/*z* 859.5617). Alterations in the ratios of seeds of *C. nodosa* for 29 compounds, recognised after the restrictive selection into the fatty acyls category, ranged from 1.0 to 1.6 (*m*/*z* 188.0899). In *P. oceanica,* the ratios reached values of 16 for a fatty acyl glycoside of mannotriose (*m*/*z* 503.1601; ppm, 3) in germinated seeds.

Regarding steroid-type compounds, it is highlighted a compound such as spironolactone (*m*/*z* 387.1991; ratio 6.5) in germinated seeds and isohydroxymethasone with *m*/*z* 389.1779 (ratio −29) for non-germinated seeds of *C. nodosa*. In *P. oceanica*, the occurrence of triterpenoid named Antcin k (*m*/*z* 487.3052; ppm, 3) was reported with a ratio 12-fold higher in germinated seeds compared with those non-germinated. Moreover, the organoheterocyclic category is mainly characterised by several types of molecules, such as pyrrolidines, indoles and benzopyrans. Benzopyrans (*m*/*z* 381.0945) of the mollicellin type were determined in germinated seeds of both seagrasses with similar ratios (2.6–2.9). In *P. oceanica*, a compound of *m*/*z* 299.0605 (ppm 4) was identified as a pyrrolidine with a ratio of 8.9-higher in germinated seeds. Apart from these types of molecules in *C. nodosa*, this category also included a molecule with a *m*/*z* 466.1158 (ppm 10) identified like thienodiazepines with a ratio 8-fold higher in germinated seeds.

Flavonoids have been identified in germinated seeds of *C. nodosa*, two and three times higher than in non-germinated seeds and in forms of quadrangularin and catechins, respectively. In the case of *Posidonia*, from 15 compounds constrained by ppm value and ratio, it is relevant the presence of anthocyanins such as cyanidin and pelargonidin derivatives (2 times higher in germinated seeds). Metabolites activated as carboxylic acids and derivatives were also encountered in similar proportions in both seeds of *C. nodosa* and *P. oceanica* (range of 2–3).

### 2.3. Marine Plant Fragment Metabolites: Assessing Antioxidant Activity in Marine Plants Washed up Coast

Polyphenols such as caffeic and coumaric acids are detected three times higher in the whole plant of *C. nodosa* compared with those of *P. oceanica*. Moreover, the highest contents of caffeic acid are quantified in mature leaves of *C. nodosa* (Table 1), while high concentrations are also reported in rhizomes and roots (Table 1). Otherwise, coumaric acid is significantly reported in the whole plant of *P. oceanica* and *C. nodosa* (Table 1) in contrast to ferulic acid, which is only detected in leaves. Gallic acid (GA) is undetected in either marine plant.

The total carbohydrate content is mainly supported by rhizomes with significant differences within other plant parts (262.50 ± 0.10 mg glucose equivalent g^−1^ dw for *P. oceanica* and 526.00 ± 1.00 mg glucose equivalent g^−1^ dw for *C. nodosa*; Table 2). Total phenolic compounds and flavonoids show significant differences in the roots and rhizomes of *P. oceanica* compared with *C. nodosa* (Table 2).

The scavenging activities of seagrasses are higher than that of the synthetic antioxidant food additive BHA (64.4%, t_1/2_ 405 s; Table 3). The inhibition percentage ranged from 80.2% to 87.3%, with the lowest t_1/2_ reported in rhizomes (87.3%, 33 s) and roots (87.0%, 38 s) for *P. oceanica*, whereas DPPH inhibition activity for *C. nodosa* corresponded to 73.70% and t_1/2_ 239 s for rhizomes and 85.2% and t_1/2_ 131 s for roots (Table 3).

Activities of reduction of Fe^3+^ and Cu^2+^ (i.e., FRAP, CUPRAC) and of Cu^2+^-chelating (CCA) are higher in rhizomes of both seagrasses compared with leaves, sheaths, roots and whole plant (Table 4). Otherwise, the lowest activities for FRAP, CUPRAC and CCA correspond to young leaves of *P. oceanica* (Table 4).

Statistical analysis in Table 5 indicates significant correlations between TPC and flavonoids for *P. oceanica* (R^2^ = 0.988, *p* < 0.001) and *C. nodosa* (R^2^ =0.963, *p* < 0.01). Positive correlations are also reported between total carbohydrates and FRAP activity (R^2^ = 0.936, *p* < 0.006) for *P. oceanica,* and with R^2^ 0.969 (*p* < 0.001) for *C. nodosa*. Likewise, total carbohydrates and CRUPAC are correlated for *P. oceanica* (R^2^ = 0.935, *p* < 0.006) and for *C. nodosa* (R^2^ 0.965 with *p* < 0.002). Additionally, TCH and TPC correlate with CCA (*p* < 0.009 and *p* < 0.014 respectively) for *C. nodosa.* No correlations for RSA are encountered.

## 3. Discussion

### 3.1. Metabolites in Seeds of Seagrasses

Seed metabolite profiles of two seagrasses exhibited different patterns, which can be explained by the distinctive reproductive strategies and seedling establishment of *P. oceanica* and *C. nodosa* plants (Figure 1). Seed differences can be attributed to the buoyant fruits of *P. oceanica*, which are transported away from the mother plant, allowing for the released seeds to trigger germination in the meantime [17,18,24]. In contrast, *Cymodocea* seeds sink, settle in the soil and continue to initiate germination.

Metabolite analysis in seeds indicated that a phospholipid pool (i.e., phosphatidylinositol, phosphatidylcholines and sphingolipids) may reflect lipid trafficking, which can act in the biogenesis of organelle membranes, signalling processes and the mobilisation of storage lipids required for seed germination in both seagrasses. These results correlate with the observation that *P. oceanica* seeds display high metabolic activity to supply carbon, nitrogen and phosphorus during early seedling growth [24,25]. In terrestrial plants, lipids have played a primary role in the initial stages of seed germination, as they provide the sugars and energy necessary for growth and development [26,27,28].

Notably, changes in the glycerophospholipid pool have also been associated with phosphate release in phosphorus-deficient terrestrial plants [29]. In *C. nodosa*, a high ratio (ratio 37.746; Table S3) for glycerophospholipid compounds suggests that changes in the phospholipid pool support the release of inorganic phosphorus in germinated seeds. The viability of ramets (fragments of rhizome, roots and leaves) in *C. nodosa* is known to be linked to the presence of phosphorus in the soil, as phosphorus is the main nutrient required to fulfil growth requirements [30]. This aspect warrants further study due to its significance for seed propagation and in vitro culture of this seagrass. Furthermore, the identification of a higher number of metabolites involved in the TCA cycle in *C. nodosa* compared with those in *P. oceanica* may suggest that mechanisms to mobilise carbon and nitrogen differ between the two marine plants. This indicates a mobilisation of TCA compounds in dormant seeds of *C. nodosa*, which do not float and germinate upon falling to the ground, in contrast to the mobilisation of fatty acyls in non-dormant seeds of *P. oceanica* that germinate before settling. Otherwise, in terrestrial plants, the contribution of fatty acyls to ecological mechanisms, such as signals for growth and interaction with microorganisms, has been reported [31]. During seed germination and development, the degradation of hydroxy fatty acids is associated with variations in seed size and seedling growth [8,32]. Importantly, hydroxy fatty acids are known within the category of signalling molecules for associations between plants and fungal populations [33]. These relationships between microorganisms and seeds suggest that a higher number of acyl metabolites in *C. nodosa* seeds could be associated with their dormant nature.

Hydroxytetracosanoic acid, the most abundant long-chain fatty acid in *C. nodosa* seeds, has been described for its neuroprotective properties and as a functional ingredient in food and cosmetic products [34]. Additionally, lipids interact with flavonoids to maintain reactive species homeostasis as germination and development of seeds bring more oxidised acyl and fewer unsaturated chains from lipids. Therefore, positive ratios between germinated and non-germinated seeds would be indicative of protection against damage caused by ageing tissues as seed development progresses.

In relation to steroids, the presence of a compound similar to methasone in non-germinated seeds (ratio −29; Table S2) of *C. nodosa* is significant, as it has been described as an anti-inflammatory and anti-cancer glucocorticoid [35]. To date, this compound has not been documented in plant metabolism, but results indicate that this metabolite might affect seed dormancy, as it appeared in non-germinated seeds. Hence, further studies on the synthesis pathway of this steroid are required, as control of its biosynthesis may be critical to the success of seed germination.

Another steroid metabolite in the seagrass *C. nodosa*, such as spironolactone (Table S3), is a recognised inhibitor of the action of the plant growth regulator brassinosteroids in plants. The presence of spironolactone causes retardation of the growth of the embryogenic axis in Arabidopsis [36]. If, as occurs in terrestrial plants, the presence of this metabolite retards hypocotyl growth in *C. nodosa*, it would suggest that seed germination could fail, indicating seed non-viability. These results suggest that steroids control hypocotyl growth at all stages of seed development (germinated and non-germinated) in *C. nodosa*. Conversely, steroids in *P. oceanica* are represented by triterpenoids, which are recognised as secondary plant metabolites with beneficial roles in plant defence against various stress types. Again, the buoyant nature of *P. oceanica* fruits and non-dormant seeds allows for the inference that metabolites can be used as distinctive markers of developing seeds compared to those of *C. nodosa* seeds.

Moreover, organoheterocyclic compounds, represented by a benzopyran structural motif and pyrrolidines, are biologically active and naturally occurring compounds reported in both seagrasses. The benzopyran core offers potential for the design of new drug-like molecules with therapeutic applications [37,38], and particularly mollicellin (Table S3), a metabolite naturally produced by plant endophytes, has been described for resistance under adverse conditions [37]. Meanwhile, indole-based compounds have demonstrated appropriate functions as defence and resistance promoters, as well as regulators of maturation and germination of seeds [39]. Pyrrolidines attract interest in in vitro cultures as they are degradation products of the catabolism of the polyamines spermidine and spermine. Polyamines have engaged effects during the growth and development of *C. nodosa*, being reported in the apical section of the rhizome and described as an anti-senescence-inducing factor [40]. Furthermore, metabolite analysis revealed the occurrence of molecules such as benzodiazepines, which modulate the synthesis of γ-aminobutyric acid (GABA), and in turn, GABA is synthesized through the polyamine metabolic pathway. GABA accumulation works in synergy for plant growth and development and plant stress tolerance [41].

All in all, metabolite analysis has unveiled a compound profile for two types of seeds (dormant vs. non-dormant) according to developmental stage (germinated vs. non-germinated). Although we cannot disentangle certain information, such as the age of seeds, burial or buoyant time and water content, among other factors, glycerophospholipids seem to support seed germination through their mobilisation and degradation in *P. oceanica*. Contrastingly, in *C. nodosa*, mobilisation seems to occur through the TCA cycle as dormant seeds need to ensure germination at the optimal time. Steroids report a differential role according to whether seeds have or not dormancy. In the case of dormant seeds such as *Cymodocea*, metabolites seem to be responsible for delaying germination by controlling brassinosteroid biosynthesis and polyamine pathways. Otherwise in non-dormant seeds, they synthetised steroids to act in defence against stresses, as occurred to *Posidonia*.

### 3.2. Marine Plant Metabolites: Assessing Antioxidant Activity in Marine Plants Washed up Coast

There is considerable evidence with respect to the importance of phenolic compounds in scavenging free radicals and preventing oxidative damage caused by different stresses [42,43]. In the present work, mature leaves of seagrasses showed the highest total content of identified polyphenols mainly due to caffeic acid reported in both plants (17.00 ± 0.02 and 98.00 ± 0.03 µg g^−1^ dw; Table 1). This could be explained as caffeic acid favours lignin synthesis that, in turn, deals with the cell wall thickness and deceleration of cell wall expansion in mature leaves [43,44,45]. In addition, caffeic tartrates have been reported as the most abundant compounds in leaves of *P. oceanica* compared with the low levels of ferulic and coumaric tartrates [46]. In young leaves of *C. nodosa*, ferulic acid can be correlated to mechanical strength as FA-mediated cross-linking between polysaccharides. Low levels of FA and cinnamic acid (Table 1) may also be associated with changes in the lignin monomer composition, as can be exemplified with FA, which results from the methylation of caffeic acid [47]. In this sense, Leri et al. [42] determined 1.79% of ferulic acid from the total of polyphenols analysed in *P. oceanica*.

Notwithstanding, RSA was higher in marine plants than in that of the commercial synthetic antioxidant BHA (64.4%) despite being used at twice the legally permitted concentration (Table 3). Moreover, RSA is intricately linked to short t_1/2,_ which refers to the time required to reduce the initial concentration of DPPH radical by 50%. The combination of RSA and t_1/2_ revealed that reasonable scavenging activity was reported in *P. oceanica* compared with *C. nodosa*, which required longer times to scavenge radicals (Table 3). Taking into consideration our results on TPC, flavonoids (Table 2) and RSA (Table 3), these would indicate that rhizomes and roots from seagrasses washed up on the coast can be used as priming to analyse new potential polyphenols as high TPC and flavonoids are associated with rhizomes and roots of both seagrasses (Table 2, Table 3, Table 4 and Table 5). Notably, TPC in *P. oceanica* ranged from 7.80 ± 0.36 to 51.57 ± 0.92 mg GA equivalent g^−1^ dw, and from 7.04 ± 0.20 to 20.70 ± 0.18 mg GA equivalent g^−1^ dw in *C. nodosa* extracts. While flavonoids in *P. oceanica* reached levels of 30.00 ± 1.00 and 42.00 ± 1.00 mg quercetin equivalent g^−1^ dw in roots and rhizomes, respectively, and ca. 13.00 ± 1.00 mg quercetin equivalent g^−1^ dw in *C. nodosa* in both roots and rhizomes (Table 2). In naturally collected seagrass species, TPC ranged from 0.29 to 14.24 mg GA equivalent g^−1^ dw and flavonoid content fluctuated from 0.091 to 24.5 mg quercetin equivalent g^−1^ dw for *Oceana serrulata* (formerly *Cymodocea serrulata*) [11,48,49].

Similarly, the total carbohydrate contents of seagrasses (118.10 ± 0.20 and 137.00 ± 00 mg glucose g^−1^ dw in *P. oceanica* and *C. nodosa*, respectively; Table 2) were comparable to those of naturally collected seagrasses (130 mg glucose g^−1^ dw for *C. nodosa* [50]). Considering plant parts, carbohydrate contents were significant in roots, sheaths and rhizomes (Table 2), consistent with the results reported by Kim et al. [51], with leaves showing the lowest contents, in agreement with data obtained by El Din and El-Sheri [52] (28.98 and 47.22 mg g^−1^ dw for *Posidonia* and *Cymodocea,* respectively).

No correlation was found between TPC and RSA (Table 5) in either of the seagrasses. This suggests the presence of other types of metabolites responsible for this antioxidant activity. However, TPC correlates with FRAP and CCA in *C. nodosa*; specifically, flavonoids correlate with the reducing and chelating activities, indicating that these compounds, along with TCH, are the main contributors to these activities, as they show the same correlations. In fact, two new prenylated flavonoids, along with others identified as catechins, have been detected in the rhizomes of *C. nodosa* [53].

Furthermore, reducing activities (FRAP and CRUPAC) correlate with TPC and TCH in *P. oceanica*, but not with flavonoids, indicating that other types of polyphenols may be responsible for this activity. In fact, chicoric and caftaric acids, which have shown high reducing capacity [54], have been identified as the most abundant phenolic compounds in *P. oceanica* [46]. The lack of correlation for radical scavenging activity can be explained by the different chemical reactions and kinetics associated with these reactions.

Despite previous reports showing that polysaccharides of seagrasses exhibit free radical scavenging activity, the biological activities of carbohydrates depend on multiple structural features, with the most important being the presence of protein moieties and covalently linked phenolic compounds. These features allow carbohydrates to exhibit RSA and metal-reducing ability, which polysaccharides devoid of phenolic and protein groups do not exhibit [55,56]. This could explain the significant correlations found between TCH and TPC and between TCH and flavonoids in *C. nodosa* (Table 5). Overall, it is assumed that *Posidonia* and *Cymodocea* are structurally different plants as changes in lignin monomer (e.g., carbohydrates and phenolic compounds and different flavonoids; Table 1, Table 2 and Table 5) and in both antioxidant activities of reduction and complexation (Table 3, Table 4 and Table 5) were obtained. Our results reinforce the idea that seagrasses washed up on the coast can be exploited, as they are potential sources of natural antioxidants with important applications for the pharmaceutical and food industries [51,57,58,59,60]. Chelation of redox-active metals can prevent their participation in the formation of Reactive Oxygen Species (ROS) and the subsequent oxidative damage that would lead to certain diseases [61,62,63,64].

## 4. Materials and Methods

### 4.1. Sampling of Seeds and Fragments Washed up on Coast

**Figure 4:**
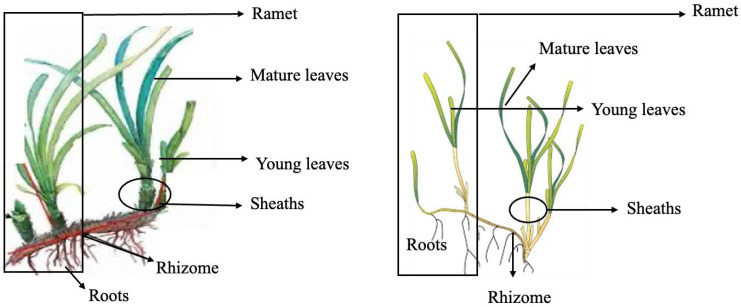
Schematic of *Posidonia oceanica* and *Cymodocea nodosa* showing parts of an individual ramet and each part of the plant.

*Posidonia oceanica* fragments and seeds were collected at 38°38.137′ N; 0°4.267′ E (Alicante, Spain) and *C. nodosa* at 27°45.208′ N; 15°40.255′ W in Gran Canaria (Canary Islands, Spain) when winter storms dragged fragments from natural meadows, i.e., 3–4 collections by season. After collection, samples were immediately stored in portable fridges at 4 °C until arrival at the laboratory. In the case of *P. oceanica*, samples were mailed to the Gran Canaria laboratory in crystal blue pearls hydrated with seawater (water crystal pearls, Amazon.co.uk [65]). To continue, sand and salt were removed by rinsing with distilled water, and samples were sorted into different parts, namely rhizome, roots, mature and young leaves, sheaths and ramets (henceforth whole plant; Figure 4).

Freeze-dried fragments (ca. 30 g for each part of the plant) were separately milled and kept in darkness at −20 °C before analysis. Pooled samples from different collections allow averaging out individual variations and minimizing the impact of fluctuations in concentrations as normalization through pooled samples maintains overall metabolite balance.

**Figure 5:**
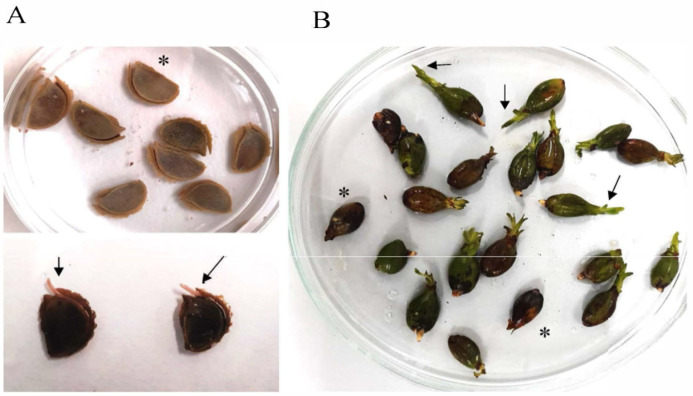
Seeds of (**A**) *Cymodocea nodosa* and (**B**) *Posidonia oceanica*. Asterisk shows non-germinated seeds, and arrows indicate small sprout for *P. oceanica* and detachment of the dorsal ridge of the *C. nodosa* seeds.

Regarding seeds and due to seed scarcity, seeds of *P. oceanica* and *C. nodosa* were both sorted into two groups, such as germinated and non-germinated seeds, for metabolomic analysis. Germinated seeds were assumed according to a morphological criterium based on the presence of a small sprout of 0.2–0.5 cm in length for *P. oceanica* and on detachment of the dorsal ridge of the *C. nodosa* seeds (Figure 5; [20]). All seeds were surface-sterilized, weighed and freeze-dried.

### 4.2. Hydrophilic Interaction Liquid Chromatography (HILIC) Coupled to Hybrid Quadrupole-Time of Flight Mass Spectrometry (QTOF-MS)-Based Metabolomic Analysis of Seeds of Two Seagrasses

Metabolomic analysis, following Gika et al. [66], was carried out with germinated and non-germinated seeds of *P. oceanica* and *C. nodosa*. Seeds (ca. 15–20 for stage) were homogenised to generate 15 mg of pooled sample, and then 300 μL methanol solution (80%) spiked with kinetin at 2 mg L^−1^ final concentration was added. The mixture was then placed in an ultrasound bath for 10 min at room temperature. To continue, the sample was centrifuged at 10,000 rpm for 10 min at 4 °C. For analysis, the supernatant was diluted 1:4 with pure acetonitrile (LCMS grade) and then filtered through PTFE filters (0.2 µm pore size). Metabolite profiling was performed using hydrophilic interaction liquid chromatography (HILIC) coupled to hybrid quadrupole-time of flight mass spectrometry (QTOF-MS). HILIC separation was performed on a 2.1 mm × 150 mm ACQUITY UPLC 1.7 µm BEH amide column (Ethylene Bridged Hybrid; Waters Corp. Ltd., Milford, MA, USA) using two-step gradients over the course of 30 min at 300 µL min^−1^. The gradient started with a 4 min isocratic step at 100% A (acetonitrile: water, 95:5 (*v*/*v*), 0.1% ammonium formate), then rising to 28% B (acetonitrile: water, 2:98 (*v*/*v*), 0.2% ammonium formate) over the next 21 min and finally to 60% B over 5 min. The injection volume was 5 µL, and the column temperature was maintained at 40 °C. A mass spectrometer is calibrated on a daily basis by infusing an aqueous solution of HCOOLi, which is acquired within the 50–1000 mass range with a resolution of 6500, rendering a residual of 0.0007 arbitrary mass units. Variations in retention time range between 0.54–1.2 s. Accurate mass value is ensured by co-infusion of a lockmass reference compound leukine enkephalin, which ensures that any mass value drift caused by environmental changes will be compensated automatically. Hence, reproducibility and correlation efficiency in each batch run is assumed.

### 4.3. Processing and Analysis of Metabolomic Data of Seagrass Seeds

Raw data were processed, and metabolites were identified through a comparison of experimental mass spectra with entries in public databases (Massbank, HMDB; [67,68]), involving manual searches for metabolites and leveraging the Galaxy platform. The relative amount of each metabolite was comparatively quantified and normalised with internal standards, such as kinetin, and with a precise weight of each pooled sample. Then, data of the relative amount of each compound were transformed to log2. For a global analysis of seed metabolites, principal component analysis (PCA) was implemented using R package version 4.2.1 (http://www.r-project.org/ (accessed on 1 November 2024)). Also, R statistical language was used for data plotting for heat maps and for plotting log2 fold-change values (*x*-axis).

To study variations of metabolites among two stages of development, namely germinated and non-germinated for each one of seagrasses, data of the normalised amount of each compound were transformed to log2, and then ratios of germinated/non-germinated for each compound of the same ratio *m*/*z* were calculated. For analysis, metabolites were selected constrained to a default 0–10 ppm mass error (mass accuracy; [69]). As a ratio of one means equal or similar proportion between germinated and non-germinated seeds, metabolites with a ratio with values below one are assumed to be part of non-germinated seed metabolism. Similarly, ratio values of each of the different metabolites were constrained to ratios over 1, and once obtained, another selection was carried out with ratios over the average value of all ≥1 ratio. These metabolites are significantly assigned to germinated-seed metabolism.

### 4.4. Marine Plant Fragment Metabolites: Assessing of Antioxidant Activity

The identification and quantification of six selected polyphenols was carried out by UHPLC-MS: GA, *p*-coumaric acid (COU), ferulic acid (FA), syringic acid (SYR), cinnamic acid (CA) and caffeic acid (CAA). For it, freeze-dried each fragment type (pooled 100 mg) was resuspended in 1 mL methanol–water (1:1) mixture, sonicated for 15 min and centrifuged. The supernatant was then filtered through a 0.21 μm nylon filter. Analyses were performed on a Thermo Orbitrap Q exactive focus mass spectrometer coupled to a Thermo Vanquish UHPLC system (Thermo Fisher Sci., Waltham, MA, USA). Measurements were performed by electrospray ionization in positive or negative mode, as required by each analyte. A chromatography column Acentis Express C-18 of 2.7 μm pore size 2.1 × 100 mm was used and eluted with water with 0.1% formic acid (solution A) and methanol (solution B) at a flow rate of 0.4 mL min^−1^. The chromatographic conditions were programmed as follows: 95% solution A and 5% solution B for 5 min. Then, a course of 2.5 min run was at 100% solution B and continued with 95% solution A and 5% solution B until 12 min. External calibration was performed for each of the compounds. The correlation coefficients were not less than 0.9995. Reproducibility was assessed using five determinations at 0.5 ng g^−1^ and expressed as relative standard deviation (RSD), which ranged from 0.9 to 6.5%. The limits of detection (LOD) and the limits of quantification (LOQ) were calculated assuming a minimum detectable signal-to-noise level of 3 and 10, respectively. LOD were found to be in the range of 0.05–0.01 ng g^−1^, and the LOQ were observed in the range of 0.1–0.03 ng g^−1^. The recoveries were found in the range of 80–110%. Standard and sample chromatograms are deposited as Supplementary Material (Table S4). To assure determination, a default 5 ppm mass error was set. All samples were assayed with two independent replicates for each fragment type. Results were reported as mean ± standard deviation (SD).

Also, each freeze-dried plant fragment (ca. 100 mg pooled each; Figure 4) was suspended in 1.5 mL of Milli-Q water, sonicated for 30 min, stirred for 30 min and centrifuged at 7000 rpm for 15 min. The supernatant was then withdrawn, and the pellet was extracted with 1.5 mL of water for 30 min and centrifuged as indicated above. Supernatants were collected and reserved at 4 °C for determining total carbohydrates and reducing antioxidant power (FRAP and CUPRAC) and Cu (II) chelating (CCA) activities. Extraction of freeze-dried plant-sorted material (ca. 100 mg each) was also carried out with 1.5 mL of methanol for phenolic contents, flavonoids and RSA activity following the same procedure described below.

The total carbohydrate content (TCH) was determined using the colorimetric method described by Brooks et al. [70] with modifications. Each aqueous extract (100 µL) was diluted with water (900 µL) and mixed with 2 mL of freshly prepared anthrone reagent (1.0 mM in 96% sulphuric acid). Then, the mixture was heated at 100 °C in a water bath for 10 min. To follow, samples were cooled in an ice bath, and the absorbance (Abs) was recorded at 620 nm on a Shimadzu UV-1800 spectrophotometer (Shimadzu Co., Kioto, Japan).

Carbohydrate content was calculated from a standard calibration curve in a concentration range from 50 to 500 µg mL^−1^ and expressed as grams equivalent of glucose per 100 g of corresponding plant fragment (% of dry biomass). All samples were assayed in triplicate with two independent replicates for each fragment type. Results were reported as mean ± standard deviation (SD).

The total phenolic content (TPC) was determined according to the Folin–Ciocalteu assay [71]. Each sample (50 μL) was mixed with 4.2 mL of distilled water, 250 µL of Folin–Ciocalteu’s reagent and 0.5 mL of sodium carbonate (20%), and the mixture was allowed to stand for 1 h in the darkness at room temperature. The Abs was measured at 765 nm using a Shimadzu 1800 UV–Vis spectrophotometer (Shimadzu Co., Kioto, Japan). The TPC was calculated from a standard calibration curve of GA in methanol (ranging from 0.10 to 1.06 mg mL^−1^), and the results were expressed as mg of GA equivalents per gram of dry plant section powder (means ± standard deviation of three measurements with two independent replicates for each fragment type).

Total flavonoid content was quantified by an aluminium chloride colorimetric assay according to Li et al. [72] with modifications. Quercetin was used as a standard to prepare the calibration curve. A hundred µL of sample or standard solution was mixed with 400 µL of 60% ethanol and 30 µL of 5% NaNO_2_ for 6 min. Then, 15 µL of 10% AlCl_3_ was added, and the mixture was allowed to react for another 6 min. The reaction was stopped by adding 400 µL of 4% NaOH and 100 µL of ethanol. The Abs was measured after 15 min at 510 nm. Total flavonoid content was expressed as mg quercetin equivalents per gram dry weight (mean ± standard deviation of three measurements with two independent replicates for each fragment type) and was calculated from a calibration curve.

To determine radical scavenging activity (RSA), each sample (30 µL of methanolic extract) was added to 1 mL of 2,2-diphenyl-1-picrylhydrazyl (free radical DPPH) solution (0.078 mM) and left for 10 min [73]. Then, Abs was measured at 515 nm, and the ability of the plant extracts to inhibit DPPH radical was expressed as inhibition percentage and calculated as follows: RSA = 100 × (1 − (Abs in the presence of sample/Abs in the absence of sample)). The capacity to scavenge DPPH radical was referenced with that of butylated hydroxyanisole (BHA) at 500 mgL^−1^. BHA is a food additive with known antioxidant activity, whose use is permitted at a maximum level of 200 mgL^−1^ [74]. The time required to reduce the initial DPPH radical concentration by 50% (*t*_1/2_) was also calculated. Measurements were assayed in triplicate with two independent replicates for each fragment type, and data were expressed as the mean ± standard deviation (SD).

Ferric Reducing Antioxidant Power (FRAP) procedure was followed according to [73] with modifications. The FRAP reagent was freshly prepared by mixing 25 mL of acetate buffer solution 0.3 M (pH 3.6), 2.5 mL of 2,3,5-triphenyltetrazolium chloride (TPTZ, 10 mM) in HCl (40 mM) and 2.5 mL of FeCl_3_·6H_2_O solution (20 mM). Each aqueous sample (50 μL) was mixed with 1.5 mL FRAP reagent and warmed at 37 °C for 10 min and later cooled in an ice bath. Absorbance was recorded at 593 nm. Results were expressed as µmol of reduced Fe^3+^ and calculated from a calibration curve designed by adding the FRAP reagent to a range of Fe^2+^ solutions (iron (II) sulphate heptahydrate) of known concentrations in the range from 0.58–4.64 mM. All samples were assayed in triplicate with two independent replicates for each fragment type. Results were reported as mean ± standard deviation (SD).

The cupric ion reducing capacities (CUPRAC) were determined using a solution at a ratio of 1:1 CuSO_4_·5H_2_O (10 mM)/neocuproine ethanolic solution (7.5 mM) in ammonium acetate buffer (1 M; [73]). A total of 570 µL of the solution was added water (1.08 mL) plus 50 μL of each plant section extract, and then mixed for 20 min. Absorbance against a blank reagent was recorded at 450 nm. A standard curve was prepared with Trolox (TR) solutions in the range of concentrations from 0.044 to 1.22 mM. The results were expressed as µmol equivalent of TR per gram of dry biomass. The estimation was carried out in triplicate with two independent replicates, and the results were averaged as mean ± standard deviation (SD).

The Cu^2+^-chelating activity (CCA) was quantified according to Saiga et al. [75] with modifications. Extract of each sample (0.25 mL) was mixed with 1 mL sodium acetate buffer (50 mM and pH 6.0) and 50 µL of CuSO_4_ (5 mM) for 30 min at room temperature. Then pyrocatechol violet (PV; 50 µL, 4 mM) was added, and the sample was left for 30 min. After that, absorbance was recorded at 632 nm. Distilled water was used as a control. The results were expressed as the inhibition percentage of PV–Cu^2+^ complex formation and calculated according to the equation: (1 − Abs_1_/Abs_0_) × 100, where Abs_0_ was the absorbance of the control, and Abs_1_ was the absorbance of each of the extracts. All samples were assayed in triplicate with two independent replicates for each fragment type. Results were reported as mean ± standard deviation (SD).

### 4.5. Chemicals

Methanol (HPLC gradient grade), FeCl_3_·6H_2_O and FeSO_4_·7H_2_O were purchased from Scharlab (Barcelona, Spain). Butylated hydroxyanisole (BHA) (analysis quality), D-glucose and aluminium chloride were supplied by Panreac (Barcelona, Spain). Ammonium acetate, sodium nitrite, epicatechin, anthrone, pyrocatechol violet (PV), Folin–Ciocalteu reagent, 1,1-diphenyl-2-picrylhydrazyl (DPPH), CuSO_4_·5H_2_O, 2,4,6-tri(2-pyridyl)-triazine (TPTZ), 2,9-dimethyl-1,10-phenanthroline (neocuproine), 6-hydroxy-2,5,7,8-tetramethyl-chroman-2-carboxylic acid (Trolox TR), copper(II) chloride and 96% ethanol of analytical quality were provided by Sigma–Aldrich Chemie (Steinheim, Germany). Sulfuric acid (97%) was provided by Honeywell (Charlotte, NC, USA). Ultrapure water was obtained from a Milli-Q system from Millipore (Bedford, MA, USA).

### 4.6. Data Analysis

Statistical comparisons of concentrations were performed using R software version 4.5.0 (https://www.r-project.org; accessed on 17 November 2023). A one-way ANOVA followed by the post hoc tests Tukey HSD and Dunnett T3 was used to detect significant differences (*p* ≤ 0.01) between plants and within corresponding sections. The statistical relationship between phenolic compound, carbohydrate and flavonoid contents and antioxidant activities was analysed through Pearson’s correlation test (tests were accepted as statistically significant with *p*-values < 0.05).

## 5. Conclusions

In conclusion, seagrasses *P. oceanica* and *C. nodosa* are rich sources of metabolites and antioxidant activities, with the possibility of benefiting from the different sections washed ashore. Seeds accumulate metabolites with different functionality depending on seed dormancy. These are involved in inorganic nutrient mobilization, defence and germination control. Also, the antioxidant capacities of the different plant fragments and their relationship with flavonoids and total carbohydrates are relevant. It is worth mentioning the extension of the study to new polyphenols and the investigation of the economic values of the wasted washed-up biomass of these two species. This study also opens a framework to analyse new metabolites responsible for the antioxidant activity in seagrasses.

## Figures and Tables

**Table 1:** Phenolic content (μg g^−1^ dw) quantified by UPLC-MS in seagrass sections from *Posidonia oceanica* and *Cymodocea nodosa*.

	Phenolic Compounds					
	CA	CAA	COU	FA	SYR	Sum
	*Posidonia oceanica*					
Mature leaves	n.d.	17.00 ± 0.02 ° *	0.12 ± 0.01	0.11 ± 0.01 ° *	0.03 ± 2 × 10^−3^ *	17.27 ± 9 × 10^−4^ ° *
Young leaves	n.d.	1.53 ± 0.02 *	0.26 ± 9 × 10^−4^ *	n.d.	n.d.	1.80 ± 0.01
Sheaths	n.d.	0.12± 0.01 *	0.11 ± 0.01 *	n.d.	0.028 ± 2 × 10^−3^ *	0.25 ± 0.02 *
Rhizomes	0.010 ± 1 × 10^−3^	0.17 ± 0.01 *	0.02. ± 0.002 *	n.d.	0.05 ± 4 × 10^−3^	0.35 ± 0.02 *
Roots	n.d.	0.28 ± 0.02 *	0.07 ± 0.01 *	n.d.	0.03 ± 1 × 10^−3^	0.39 ± 0.02 *
Whole plant	0.097 ± 9 × 10^−3^	0.01 ± 9 × 10^−4^ *	0.43 ± 0.04 ° *	n.d.	0.07 ± 3 × 10^−3^	0.62 ± 0.02 *
	*Cymodocea nodosa*					
Mature leaves	n.d.	98.00 ± 0.03 °	0.14 ± 0.01	n.d.	n.d.	98.14 ± 0.02
Young leaves	0.48 ± 0.01	0.26 ± 0.01	0.50 ± 0.02	0.30 ± 0.01 ° *	0.04 ± 9.8 × 10^−4^ *	1.58 ± 0.01 °
Sheaths	0.07 ± 8.6 × 10^−4^	0.22 ± 0.01	0.36 ± 0.02	0.08 ± 7 × 10^−3^ *	n.d.	0.73 ± 0.03
Rhizomes	n.d.	0.69 ± 0.02	0.43 ± 0.02	n.d.	0.06 ± 3 × 10^−3^	1.17 ± 0.06
Roots	n.d.	0.54 ± 0.03	0.25 ± 0.01	n.d.	0.03 ± 0.001	0.82 ± 0.07
Whole plant	n.d.	0.04 ± 1 × 10^−3^	1.44 ± 0.02 °	n.d.	0.06 ± 2 × 10^−3^ °	1.54 ± 0.21

* Significant differences (*p* ≤ 0.01) between *Posidonia* and *Cymodocea*; ° Significant differences (*p* ≤ 0.01) within plant. Data are a result of pooled samples for each part of plant; *n* = 2 independent measurements (mean ± standard deviation (SD)). CA, cinnamic acid; CAA, caffeic acid; COU, *p*-coumaric acid; FA, ferulic acid; SYR, syringic acid; sum means total contents of phenols in each corresponding plant fraction. n.d., non-determined.

**Table 2:** Total carbohydrates (mg glucose equivalent g^−1^ dw) and total phenolic (mg gallic acid equivalent g^−1^ dw) and flavonoid contents (mg quercetin equivalent g^−1^ dw) of different seagrass sections (mature and young leaves, sheaths, rhizomes and roots and whole plant) from *Posidonia oceanica* and *Cymodocea nodosa*.

Seagrass Part	*Posidonia oceanica*			*Cymodocea nodosa*		
	TCH	TPC	FLAV	TCH	TPC	FLAV
Mature leaves	26.97 ± 0.09	10.76 ± 0.12	7.10 ± 0.50	44.10 ± 0.10	7.04 ± 0.20	3.38 ± 0.01
Young leaves	9.73 ± 0.07 *	7.80 ± 0.36	2.50 ± 0.40	44.40 ± 0.20	12.77 ± 0.40	7.40 ± 0.60
Sheaths	212.30 ± 0.08 *	17.82 ± 0.26	9.10 ± 0.40	111.90 ± 0.40	14.00 ± 0.06	6.50 ± 0.60
Rhizomes	262.50 ± 0.10 ° *	51.57 ± 0.92 *	42.00 ± 1.00 ° *	526.00 ± 1.00	20.70 ± 0.18	13.00 ± 1.00
Roots	60.70 ± 0.30 *	32.70 ± 1.27 *	30.00 ± 1.00 *	283.00 ± 1.00	18.21 ± 0.44	13.00 ± 1.00
Whole plant	118.10 ± 0.20 *	14.00 ± 0.21	8.20 ± 0.50	137.00 ± 00	13.65 ± 0.06	8.80 ± 0.30

* Significant differences (*p* ≤ 0.01) between *Posidonia oceanica* and *Cymodocea nodosa*; ° significant differences (*p* ≤ 0.01) within plant parts. Data are mean ± standard deviation (SD) from three measurements with two independent replicates each. TCH, total carbohydrates; TPC, total phenolic content; FLAV, flavonoids.

**Table 3:** Radical scavenging activities (RSA, inhibition %) and t_1/2_ (s) for extracts from plant sections of *Posidonia oceanica* and *Cymodocea nodosa* and for synthetic compound BHA.

Seagrass Part	*Posidonia oceanica*		*Cymodocea nodosa*	
	RSA	*t*_1/2_	RSA	*t*_1/2_
Mature leaves	80.20 ± 0.50	107.00 ± 6.00	65.00 ± 1.00	309.00 ± 2.00
Young leaves	45.10 ± 0.30	923.00 ± 53.00	70.80 ± 0.60	249.00 ± 4.00
Sheaths	84.10 ± 0.30	80.00 ± 4.00	84.30 ± 0.10	138.00 ± 4.00
Rhizomes	87.30 ± 00	33.00 ± 00	73.70 ± 0.90	239.00 ± 19.00
Roots	87.00 ± 0.03	38.00 ± 00	85.20 ± 0.20	131.00
Whole plant	85.90 ± 0.64	61.00 ± 6.00	73.00 ± 5.00	267.00 ± 2.00
BHA (0.5 mg mL^−1^)	64.40 ± 1.50	405.00 ± 6.00		

Data are mean ± standard deviation (SD) from three measurements with two independent replicates each. BHA, butylated hydroxyanisole.

**Table 4:** Antioxidant activities, namely FRAP (µmol of reduced Fe (III) g^−1^ dw), CUPRAC (µmol equivalent of Trolox g^−1^ dw) and CCA (inhibition percentage of PV–Cu^2+^ complex formation) of extracts derived from *Posidonia oceanica* and *Cymodocea nodosa* sections.

Seagrass Part	*Posidonia oceanica*			*Cymodocea nodosa*		
	FRAP	CUPRAC	CCA	FRAP	CUPRAC	CCA
Mature leaves	40.30 ± 0.50	45.00 ± 2.00	70.89 ± 8 × 10^−3^	45.70 ± 0.40	51.90 ± 0.60	51.50 ± 0.40
Young leaves	26.40 ± 8 × 10^−3^	35.20 ± 0.40	66.30 ± 0.60	37.60 ± 0.40	37.80 ± 0.40	51.30 ± 0.60
Sheaths	173.00 ± 1.00 *	144.90 ± 0.10 *	83.50 ± 0.40 *	40.22 ± 0.02	42.00 ± 1.00	62.40 ± 0.90
Rhizomes	234.20 ± 0.62 °	199.00 ± 1.00 °	87.10 ± 0.30 °	222.90 ± 0.40 °	181.00 ± 2.00 °	93.60 ± 0.20
Roots	121.00 ± 0.60	116.00 ± 5.00	44.00 ± 0.90 *	150.00 ± 0.10	133.40 ± 0.30	90.40 ± 0.30
Whole plant	79.70 ± 0.05	96.00 ± 1.00	79.80 ± 0.80	39.90 ± 0.10	50.70 ± 0. 60	69.00 ± 0.10

* Significant differences (*p* ≤ 0.01) between *Posidonia oceanica* and *Cymodocea nodosa*; ° significant differences (*p* ≤ 0.01) within plant parts. Data are mean ± standard deviation (SD) from three measurements with two independent replicates each.

**Table 5:** Statistical relationship between the data of carbohydrate, polyphenol and flavonoid contents and antioxidant activities of *Posidonia oceanica* and *Cymodocea nodosa* through the Pearson correlation test.

Assay	Correlation Coefficients	*Posidonia oceanica*			*Cymodocea nodosa*		
		TCH	TPC	FLAV	TCH	TPC	FLAV
TCH	Pearson’s R	1.0		0.569	1.0		0.863 *
	*p*-value	-		0.238	-		0.027
TPC	Pearson’s R	0.678	1.0	0.988 ***	0.863 *	1.0	0.963 **
	*p*-value	0.139		<0.001	0.027		0.002
RSA	Pearson’s R	0.563	0.527	0.527	0.266	0.602	0.488
	*p*-value	0.245	0.283	0.279	0.610	0.103	0.327
FRAP	Pearson’s R	0.936 **	0.854 *	0.779	0.969 **	0.814 *	0.841 *
	*p*-value	0.006	0.030	0.068	0.001	0.049	0.036
CUPRAC	Pearson’s R	0.935 **	0.865 *	0.794	0.965 **	0.805	0.847 *
	*p*-value	0.006	0.026	0.059	0.002	0.054	0.033
CCA	Pearson’s R	0.690	0.099	−0.042	0.921 **	0.902 *	0.936 **
	*p*-value	0.130	0.852	0.936	0.009	0.014	0.006

* *p* < 0.05, ** *p* < 0.01, ***p < 0.001; TCH, total carbohydrates; TPC, total phenolic content; FLAV, flavonoids; RSA, radical scavenging activity; FRAP, Ferric Reducing Antioxidant Power; CUPRAC, cupric ion reducing capacities; CCA, Cu^2+^-chelating activity.

## Data Availability

All data have been supplied in the results and Supplementary Material of this paper.

## Supplementary Materials

The following supporting information can be downloaded at: https://www.mdpi.com/article/10.3390/md23050193/s1, Table S1. Metabolite data using the estimated compound mass [M] in modeposirtive [M + H]^+^; Table S2. Metabolite data using the estimated compound mass [M] in mode negative [M − H]^−^; Table S3. Metabolite data using the estimated compound mass [M] in mode postive [M + H]^+^ and negative [M − H]^−^ categorised in glycerolipids, OrganHeterocyclics, Fatty acyls (LIPIDS), flavonoids, steroids, compounds involved in activation processes; Table S4. Chromatogram for standard of each polyphenol analised through UHPLC-MS.
