# Supplementary material for: Metabolite Profiling and Antioxidant Activities in Seagrass Biomass
Source: Mar Drugs. 2025 Apr 29;23(5):193. doi: 10.3390/md23050193 (PMC12113406; doi:10.3390/md23050193)
Supplement: Supplementary file 1 [file marinedrugs-23-00193-s001.zip › Table S4.pdf]

Table S4

Chromatogram for standard of each polyphenol analised through UHPLC -MS.

Polyphenols are gallic acid (GA), p-coumaric acid (COU), ferulic acid (FA), syringic acid (SYR), cinnamic acid (CA) and caffeic acid (CAA).

Reproducibility was assessed using five determinations at 0,5 ng g-1and expressed as relative standard deviation (RSD).

Identification of polyphenols in different samples are exemplified with a run.

For P.oceanica, sample 1 corresponds to whole plant; 2, roots; 3, mature leaves; 4, sheaths; 5, young leaves and 6, rhizomes. For C. nodosa, the whole plant is sample 7; 8, roots; 9, rhizome; 10, mature leaves; 11, sheaths and 12 young leaves

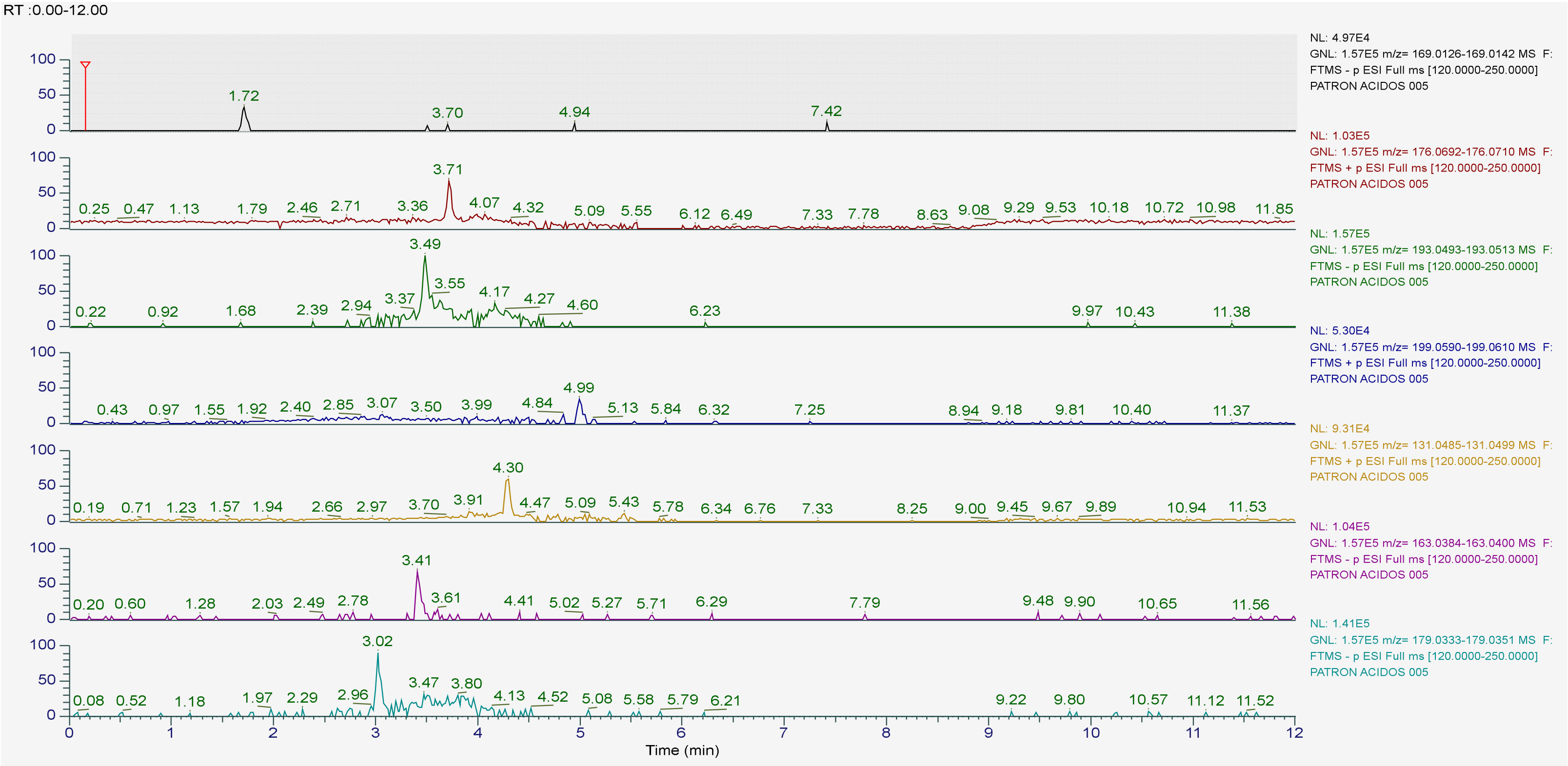
RT :0.00-12.00
NL: 4.97E4
GNL: 1.57E5 m/z= 169.0126-169.0142 MS F: FTMS - p ESI Full ms [120.0000-250.0000]
PATRON ACIDOS 005
1.72 3.70 4.94 7.42
NL: 1.03E5
GNL: 1.57E5 m/z= 176.0692-176.0710 MS F: FTMS + p ESI Full ms [120.0000-250.0000]
PATRON ACIDOS 005
0.25 0.47 1.13 1.79 2.46 2.71 3.36 3.71 4.07 4.32 5.09 5.55 6.12 6.49 7.33 7.78 8.63 9.08 9.29 9.53 10.18 10.72 10.98 11.85
NL: 1.57E5
GNL: 1.57E5 m/z= 193.0493-193.0513 MS F: FTMS - p ESI Full ms [120.0000-250.0000]
PATRON ACIDOS 005
0.22 0.92 1.68 2.39 2.94 3.37 3.49 3.55 4.17 4.27 4.60 6.23 9.97 10.43 11.38
NL: 5.30E4
GNL: 1.57E5 m/z= 199.0590-199.0610 MS F: FTMS + p ESI Full ms [120.0000-250.0000]
PATRON ACIDOS 005
0.43 0.97 1.55 1.92 2.40 2.85 3.07 3.50 3.99 4.84 4.99 5.13 5.84 6.32 7.25 8.94 9.18 9.81 10.40 11.37
NL: 9.31E4
GNL: 1.57E5 m/z= 131.0485-131.0499 MS F: FTMS + p ESI Full ms [120.0000-250.0000]
PATRON ACIDOS 005
0.19 0.71 1.23 1.57 1.94 2.66 2.97 3.70 3.91 4.30 4.47 5.09 5.43 5.78 6.34 6.76 7.33 8.25 9.00 9.45 9.67 9.89 10.94 11.53
NL: 1.04E5
GNL: 1.57E5 m/z= 163.0384-163.0400 MS F: FTMS - p ESI Full ms [120.0000-250.0000]
PATRON ACIDOS 005
0.20 0.60 1.28 2.03 2.49 2.78 3.41 3.61 4.41 5.02 5.27 5.71 6.29 7.79 9.48 9.90 10.65 11.56
NL: 1.41E5
GNL: 1.57E5 m/z= 179.0333-179.0351 MS F: FTMS - p ESI Full ms [120.0000-250.0000]
PATRON ACIDOS 005
0.08 0.52 1.18 1.97 2.29 2.96 3.02 3.47 3.80 4.13 4.52 5.08 5.58 5.79 6.21 9.22 9.80 10.57 11.12 11.52
100
50
0
0 1 2 3 4 5 6 7 8 9 10 11 12
Time (min)

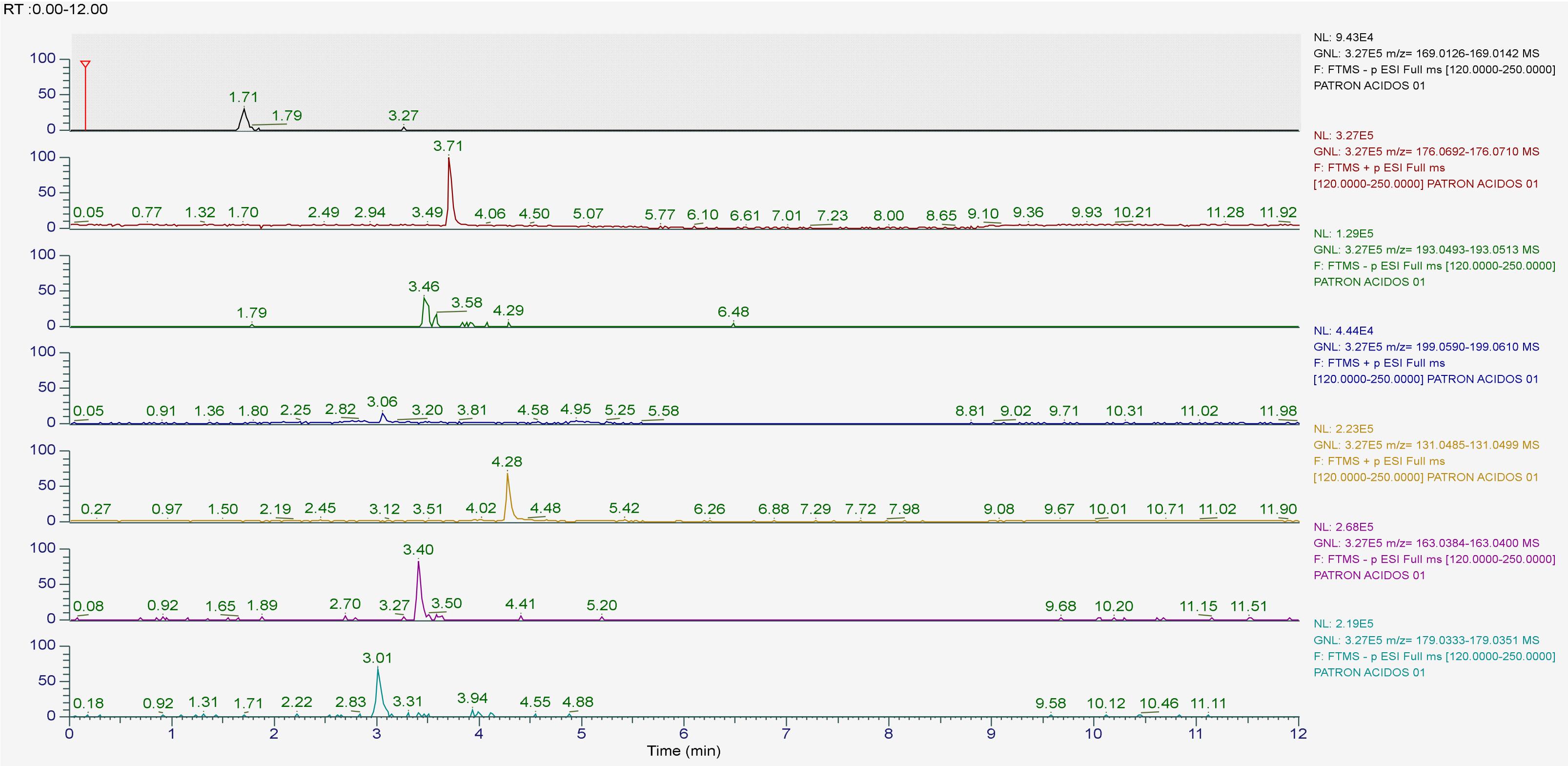

RT :0.00-12.00
100
50
0
1.71 1.79 3.27
3.71
0.05 0.77 1.32 1.70 2.49 2.94 3.49 4.06 4.50 5.07 5.77 6.10 6.61 7.01 7.23 8.00 8.65 9.10 9.36 9.93 10.21 11.28 11.92
3.46 3.58 1.79 4.29 6.48
3.06
0.05 0.91 1.36 1.80 2.25 2.82 3.20 3.81 4.58 4.95 5.25 5.58 8.81 9.02 9.71 10.31 11.02 11.98
4.28
0.27 0.97 1.50 2.19 2.45 3.12 3.51 4.02 4.48 5.42 6.26 6.88 7.29 7.72 7.98 9.08 9.67 10.01 10.71 11.02 11.90
3.40
0.08 0.92 1.65 1.89 2.70 3.27 3.50 4.41 5.20 9.68 10.20 11.15 11.51
3.01
0.18 0.92 1.31 1.71 2.22 2.83 3.31 3.94 4.55 4.88 9.58 10.12 10.46 11.11
0 1 2 3 4 5 6 7 8 9 10 11 12
Time (min)
NL: 9.43E4
GNL: 3.27E5 m/z= 169.0126-169.0142 MS
F: FTMS - p ESI Full ms [120.0000-250.0000]
PATRON ACIDOS 01
NL: 3.27E5
GNL: 3.27E5 m/z= 176.0692-176.0710 MS
F: FTMS + p ESI Full ms
[120.0000-250.0000] PATRON ACIDOS 01
NL: 1.29E5
GNL: 3.27E5 m/z= 193.0493-193.0513 MS
F: FTMS - p ESI Full ms [120.0000-250.0000]
PATRON ACIDOS 01
NL: 4.44E4
GNL: 3.27E5 m/z= 199.0590-199.0610 MS
F: FTMS + p ESI Full ms
[120.0000-250.0000] PATRON ACIDOS 01
NL: 2.23E5
GNL: 3.27E5 m/z= 131.0485-131.0499 MS
F: FTMS + p ESI Full ms
[120.0000-250.0000] PATRON ACIDOS 01
NL: 2.68E5
GNL: 3.27E5 m/z= 163.0384-163.0400 MS
F: FTMS - p ESI Full ms [120.0000-250.0000]
PATRON ACIDOS 01
NL: 2.19E5
GNL: 3.27E5 m/z= 179.0333-179.0351 MS
F: FTMS - p ESI Full ms [120.0000-250.0000]
PATRON ACIDOS 01

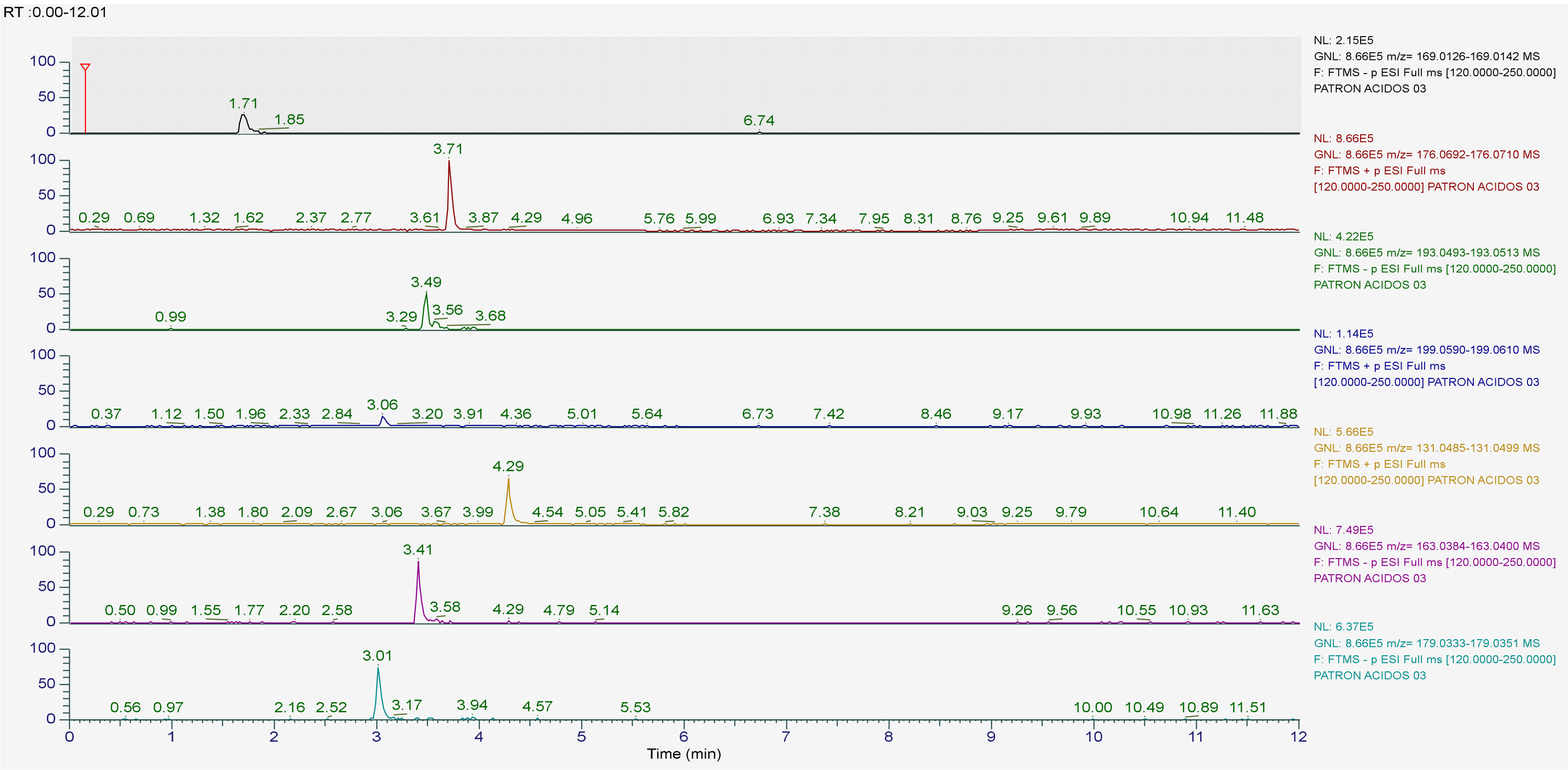

RT :0.00-12.01
NL: 2.15E5
GNL: 8.66E5 m/z= 169.0126-169.0142 MS
F: FTMS - p ESI Full ms [120.0000-250.0000]
PATRON ACIDOS 03
1.71
1.85
6.74
NL: 8.66E5
GNL: 8.66E5 m/z= 176.0692-176.0710 MS
F: FTMS + p ESI Full ms
[120.0000-250.0000] PATRON ACIDOS 03
3.71
0.29 0.69 1.32 1.62 2.37 2.77 3.61 3.87 4.29 4.96 5.76 5.99 6.93 7.34 7.95 8.31 8.76 9.25 9.61 9.89 10.94 11.48
NL: 4.22E5
GNL: 8.66E5 m/z= 193.0493-193.0513 MS
F: FTMS - p ESI Full ms [120.0000-250.0000]
PATRON ACIDOS 03
3.49
0.99 3.29 3.56 3.68
NL: 1.14E5
GNL: 8.66E5 m/z= 199.0590-199.0610 MS
F: FTMS + p ESI Full ms
[120.0000-250.0000] PATRON ACIDOS 03
3.06
0.37 1.12 1.50 1.96 2.33 2.84 3.20 3.91 4.36 5.01 5.64 6.73 7.42 8.46 9.17 9.93 10.98 11.26 11.88
NL: 5.66E5
GNL: 8.66E5 m/z= 131.0485-131.0499 MS
F: FTMS + p ESI Full ms
[120.0000-250.0000] PATRON ACIDOS 03
4.29
0.29 0.73 1.38 1.80 2.09 2.67 3.06 3.67 3.99 4.54 5.05 5.41 5.82 7.38 8.21 9.03 9.25 9.79 10.64 11.40
NL: 7.49E5
GNL: 8.66E5 m/z= 163.0384-163.0400 MS
F: FTMS - p ESI Full ms [120.0000-250.0000]
PATRON ACIDOS 03
3.41
0.50 0.99 1.55 1.77 2.20 2.58 3.58 4.29 4.79 5.14 9.26 9.56 10.55 10.93 11.63
NL: 6.37E5
GNL: 8.66E5 m/z= 179.0333-179.0351 MS
F: FTMS - p ESI Full ms [120.0000-250.0000]
PATRON ACIDOS 03
3.01
0.56 0.97 2.16 2.52 3.17 3.94 4.57 5.53 10.00 10.49 10.89 11.51
100
50
0
0 1 2 3 4 5 6 7 8 9 10 11 12
Time (min)

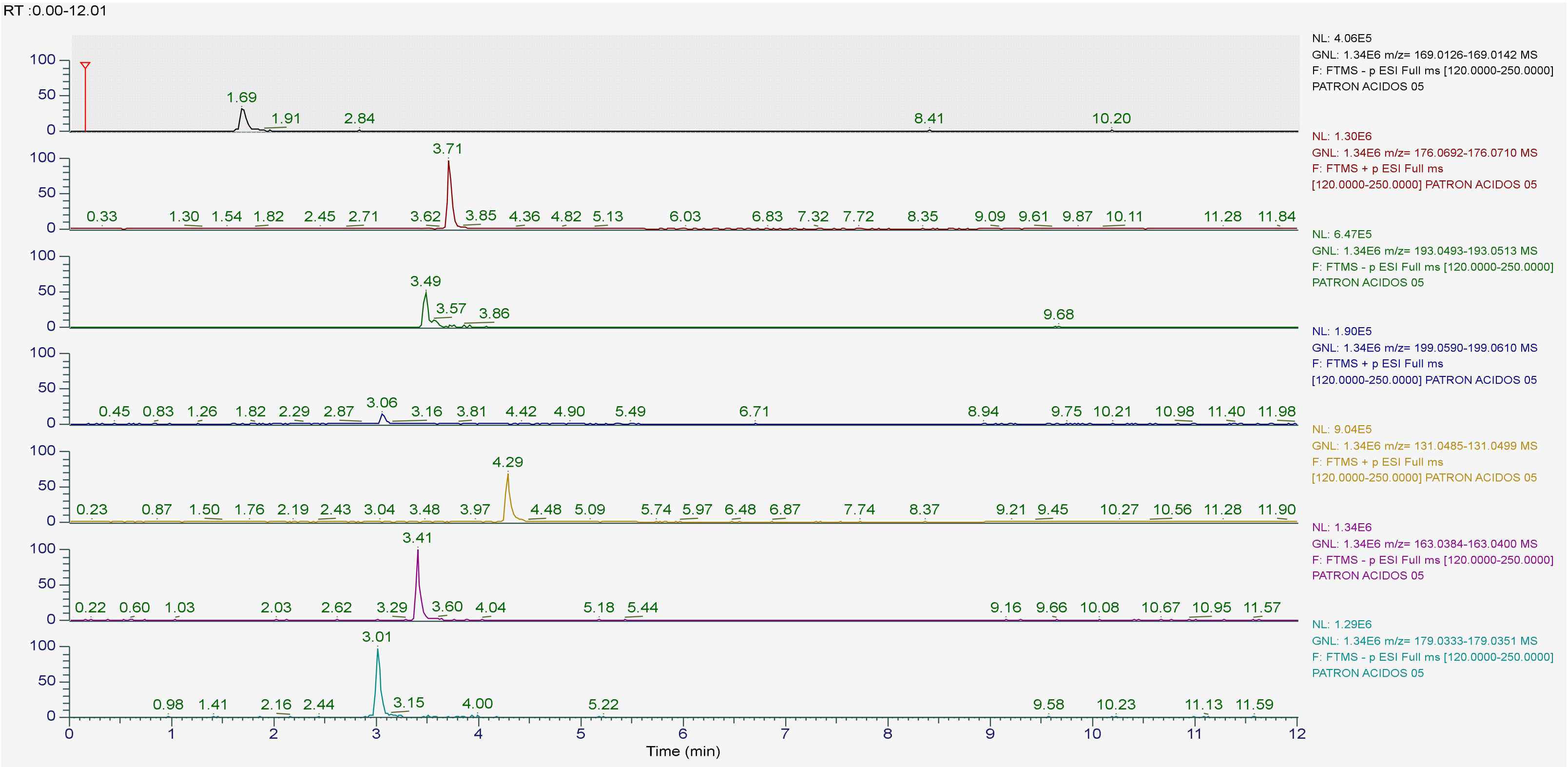

RT :0.00-12.01
NL: 4.06E5
GNL: 1.34E6 m/z= 169.0126-169.0142 MS
F: FTMS - p ESI Full ms [120.0000-250.0000]
PATRON ACIDOS 05
NL: 1.30E6
GNL: 1.34E6 m/z= 176.0692-176.0710 MS
F: FTMS + p ESI Full ms
[120.0000-250.0000] PATRON ACIDOS 05
NL: 6.47E5
GNL: 1.34E6 m/z= 193.0493-193.0513 MS
F: FTMS - p ESI Full ms [120.0000-250.0000]
PATRON ACIDOS 05
NL: 1.90E5
GNL: 1.34E6 m/z= 199.0590-199.0610 MS
F: FTMS + p ESI Full ms
[120.0000-250.0000] PATRON ACIDOS 05
NL: 9.04E5
GNL: 1.34E6 m/z= 131.0485-131.0499 MS
F: FTMS + p ESI Full ms
[120.0000-250.0000] PATRON ACIDOS 05
NL: 1.34E6
GNL: 1.34E6 m/z= 163.0384-163.0400 MS
F: FTMS - p ESI Full ms [120.0000-250.0000]
PATRON ACIDOS 05
NL: 1.29E6
GNL: 1.34E6 m/z= 179.0333-179.0351 MS
F: FTMS - p ESI Full ms [120.0000-250.0000]
PATRON ACIDOS 05
Time (min)

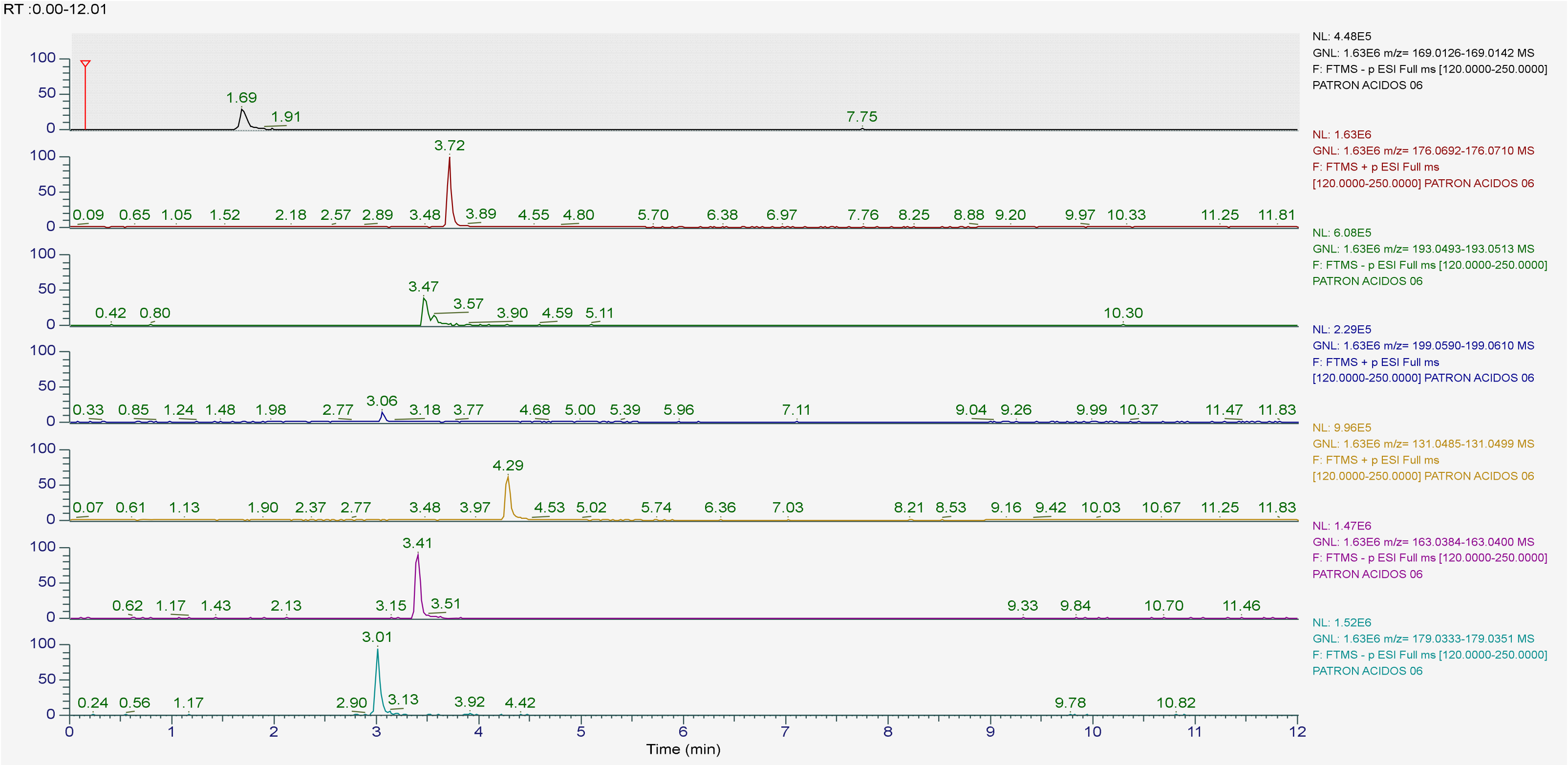

RT :0.00-12.01
NL: 4.48E5
GNL: 1.63E6 m/z= 169.0126-169.0142 MS
F: FTMS - p ESI Full ms [120.0000-250.0000]
PATRON ACIDOS 06
NL: 1.63E6
GNL: 1.63E6 m/z= 176.0692-176.0710 MS
F: FTMS + p ESI Full ms
[120.0000-250.0000] PATRON ACIDOS 06
NL: 6.08E5
GNL: 1.63E6 m/z= 193.0493-193.0513 MS
F: FTMS - p ESI Full ms [120.0000-250.0000]
PATRON ACIDOS 06
NL: 2.29E5
GNL: 1.63E6 m/z= 199.0590-199.0610 MS
F: FTMS + p ESI Full ms
[120.0000-250.0000] PATRON ACIDOS 06
NL: 9.96E5
GNL: 1.63E6 m/z= 131.0485-131.0499 MS
F: FTMS + p ESI Full ms
[120.0000-250.0000] PATRON ACIDOS 06
NL: 1.47E6
GNL: 1.63E6 m/z= 163.0384-163.0400 MS
F: FTMS - p ESI Full ms [120.0000-250.0000]
PATRON ACIDOS 06
NL: 1.52E6
GNL: 1.63E6 m/z= 179.0333-179.0351 MS
F: FTMS - p ESI Full ms [120.0000-250.0000]
PATRON ACIDOS 06
1.69 1.91 7.75
3.72 0.09 0.65 1.05 1.52 2.18 2.57 2.89 3.48 3.89 4.55 4.80 5.70 6.38 6.97 7.76 8.25 8.88 9.20 9.97 10.33 11.25 11.81
3.47 3.57 0.42 0.80 3.90 4.59 5.11 10.30
3.06 0.33 0.85 1.24 1.48 1.98 2.77 3.18 3.77 4.68 5.00 5.39 5.96 7.11 9.04 9.26 9.99 10.37 11.47 11.83
4.29 0.07 0.61 1.13 1.90 2.37 2.77 3.48 3.97 4.53 5.02 5.74 6.36 7.03 8.21 8.53 9.16 9.42 10.03 10.67 11.25 11.83
3.41 0.62 1.17 1.43 2.13 3.15 3.51 9.33 9.84 10.70 11.46
3.01 0.24 0.56 1.17 2.90 3.13 3.92 4.42 9.78 10.82
Time (min)

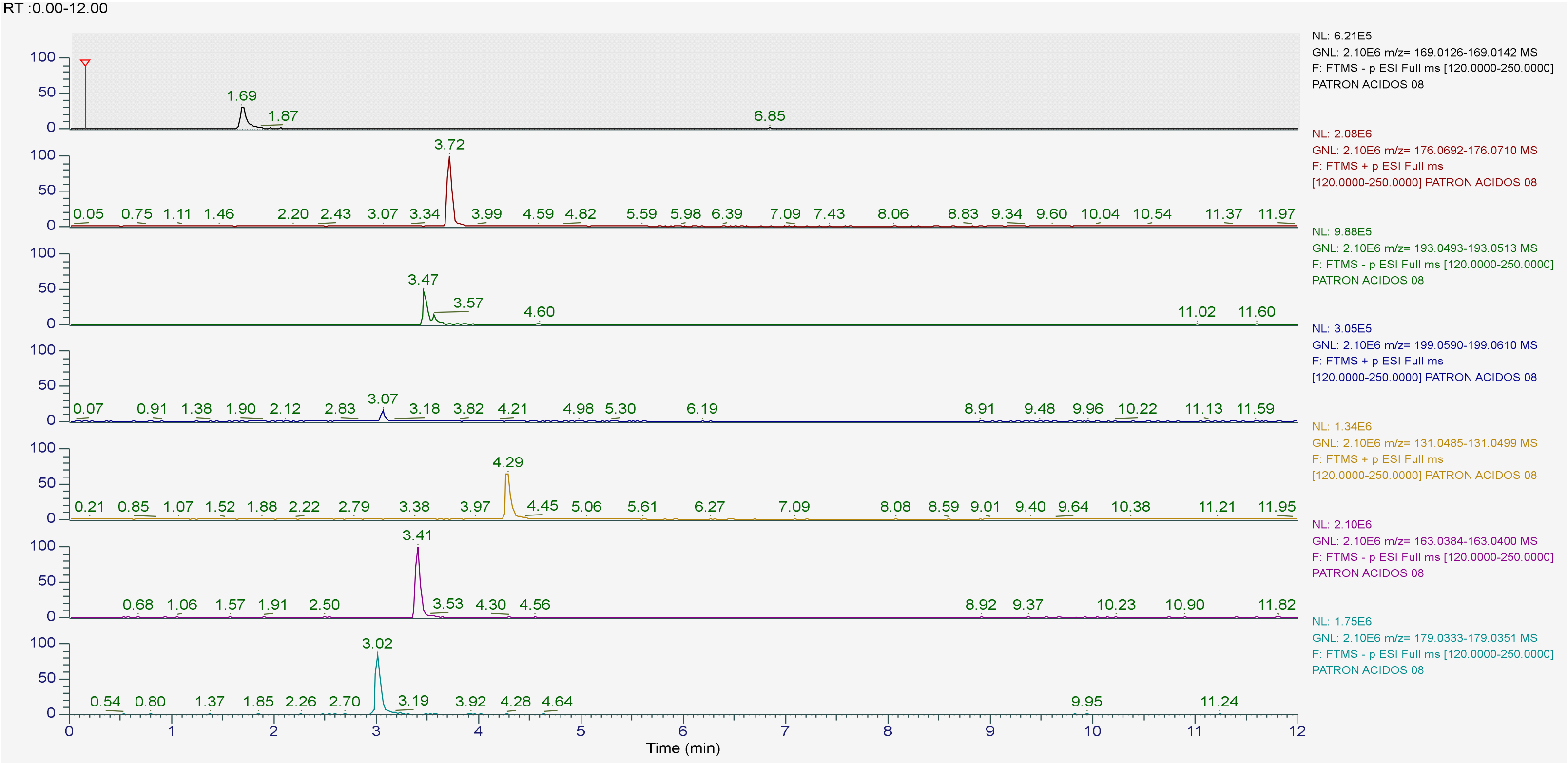
RT :0.00-12.00
NL: 6.21E5
GNL: 2.10E6 m/z= 169.0126-169.0142 MS
F: FTMS - p ESI Full ms [120.0000-250.0000]
PATRON ACIDOS 08
NL: 2.08E6
GNL: 2.10E6 m/z= 176.0692-176.0710 MS
F: FTMS + p ESI Full ms
[120.0000-250.0000] PATRON ACIDOS 08
NL: 9.88E5
GNL: 2.10E6 m/z= 193.0493-193.0513 MS
F: FTMS - p ESI Full ms [120.0000-250.0000]
PATRON ACIDOS 08
NL: 3.05E5
GNL: 2.10E6 m/z= 199.0590-199.0610 MS
F: FTMS + p ESI Full ms
[120.0000-250.0000] PATRON ACIDOS 08
NL: 1.34E6
GNL: 2.10E6 m/z= 131.0485-131.0499 MS
F: FTMS + p ESI Full ms
[120.0000-250.0000] PATRON ACIDOS 08
NL: 2.10E6
GNL: 2.10E6 m/z= 163.0384-163.0400 MS
F: FTMS - p ESI Full ms [120.0000-250.0000]
PATRON ACIDOS 08
NL: 1.75E6
GNL: 2.10E6 m/z= 179.0333-179.0351 MS
F: FTMS - p ESI Full ms [120.0000-250.0000]
PATRON ACIDOS 08
1.69
3.72
3.47
3.07
4.29
3.41
3.02
Time (min)

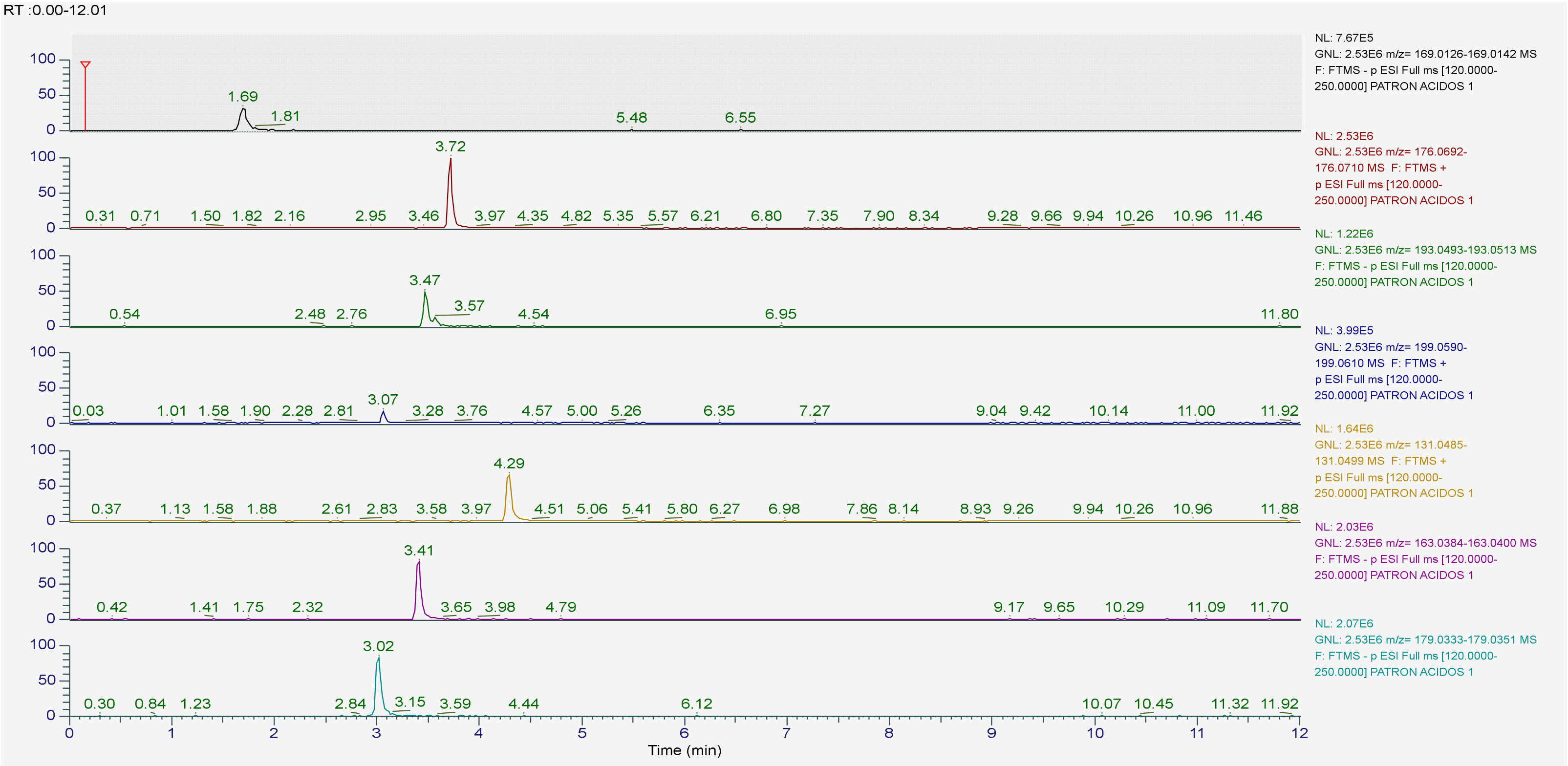

RT :0.00-12.01
NL: 7.67E5
GNL: 2.53E6 m/z= 169.0126-169.0142 MS
F: FTMS - p ESI Full ms [120.0000-250.0000] PATRON ACIDOS 1
NL: 2.53E6
GNL: 2.53E6 m/z= 176.0692-176.0710 MS F: FTMS + p ESI Full ms [120.0000-250.0000] PATRON ACIDOS 1
NL: 1.22E6
GNL: 2.53E6 m/z= 193.0493-193.0513 MS
F: FTMS - p ESI Full ms [120.0000-250.0000] PATRON ACIDOS 1
NL: 3.99E5
GNL: 2.53E6 m/z= 199.0590-199.0610 MS F: FTMS + p ESI Full ms [120.0000-250.0000] PATRON ACIDOS 1
NL: 1.64E6
GNL: 2.53E6 m/z= 131.0485-131.0499 MS F: FTMS + p ESI Full ms [120.0000-250.0000] PATRON ACIDOS 1
NL: 2.03E6
GNL: 2.53E6 m/z= 163.0384-163.0400 MS
F: FTMS - p ESI Full ms [120.0000-250.0000] PATRON ACIDOS 1
NL: 2.07E6
GNL: 2.53E6 m/z= 179.0333-179.0351 MS
F: FTMS - p ESI Full ms [120.0000-250.0000] PATRON ACIDOS 1
Time (min)

GALICO_GA

1767949,5		
1878330,09		
1633884,36		
1667273,04		
1791879,48		
PROMEDIO	DESVIACION	RSD
1747863,29	98550,0541	5,6

INDOLACETICO_IAA

6735142,51		
6538589,25		
6628410,85		
6687263,5		
6740191,11		
PROMEDIO	DESVIACION	RSD
6665919,44	84237,0991	1,3

TRANS FERULICO_ FA

1964909,7		
1698927,96		
1720535,97		
1752829,9		
1708718,16		
PROMEDIO	DESVIACION	RSD
1769184,34	111281,922	6,3

SIRINGICO_SYR

536876,235		
528057,028		
530097,199		
534584,062		
524415,599		
PROMEDIO	DESVIACION	RSD
530806,025	5000,44934	0,9

TRANS CINAMICO_CA

2047950,55		
2071725,59		
2054276,93		
2035954,15		
2280614,49		
PROMEDIO	DESVIACION	RSD
2098104,34	102840,512	4,9

PARA CUMARICO_COU

3582064,52		
3362181,79		
3839797,21		
3840118,53		
3377153,39		
PROMEDIO	DESVIACION	RSD
3600263,09	235424,649	6,5

CAFEICO_CAA

4011794,08		
4004952,23		
4094641,68		
4117981,23		
4065692,13		
PROMEDIO	DESVIACION	RSD
4059012,27	49858,4465	1,2

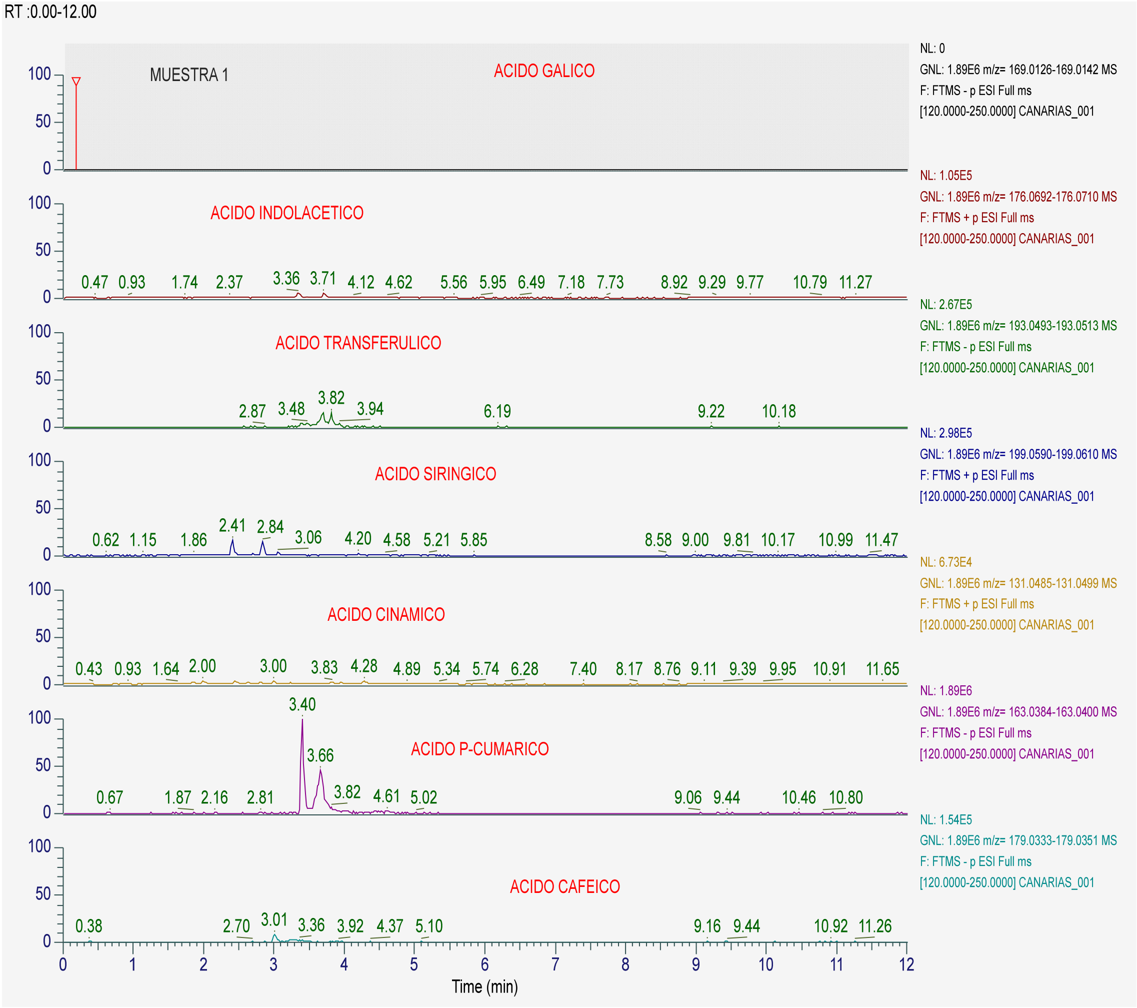
RT :0.00-12.00
MUESTRA 1
ACIDO GALICO
NL: 0
GNL: 1.89E6 m/z= 169.0126-169.0142 MS
F: FTMS - p ESI Full ms
[120.0000-250.0000] CANARIAS_001
ACIDO INDOLACETICO
NL: 1.05E5
GNL: 1.89E6 m/z= 176.0692-176.0710 MS
F: FTMS + p ESI Full ms
[120.0000-250.0000] CANARIAS_001
ACIDO TRANSFERULICO
NL: 2.67E5
GNL: 1.89E6 m/z= 193.0493-193.0513 MS
F: FTMS - p ESI Full ms
[120.0000-250.0000] CANARIAS_001
ACIDO SIRINGICO
NL: 2.98E5
GNL: 1.89E6 m/z= 199.0590-199.0610 MS
F: FTMS + p ESI Full ms
[120.0000-250.0000] CANARIAS_001
ACIDO CINAMICO
NL: 6.73E4
GNL: 1.89E6 m/z= 131.0485-131.0499 MS
F: FTMS + p ESI Full ms
[120.0000-250.0000] CANARIAS_001
ACIDO P-CUMARICO
NL: 1.89E6
GNL: 1.89E6 m/z= 163.0384-163.0400 MS
F: FTMS - p ESI Full ms
[120.0000-250.0000] CANARIAS_001
ACIDO CAFEICO
NL: 1.54E5
GNL: 1.89E6 m/z= 179.0333-179.0351 MS
F: FTMS - p ESI Full ms
[120.0000-250.0000] CANARIAS_001
Time (min)

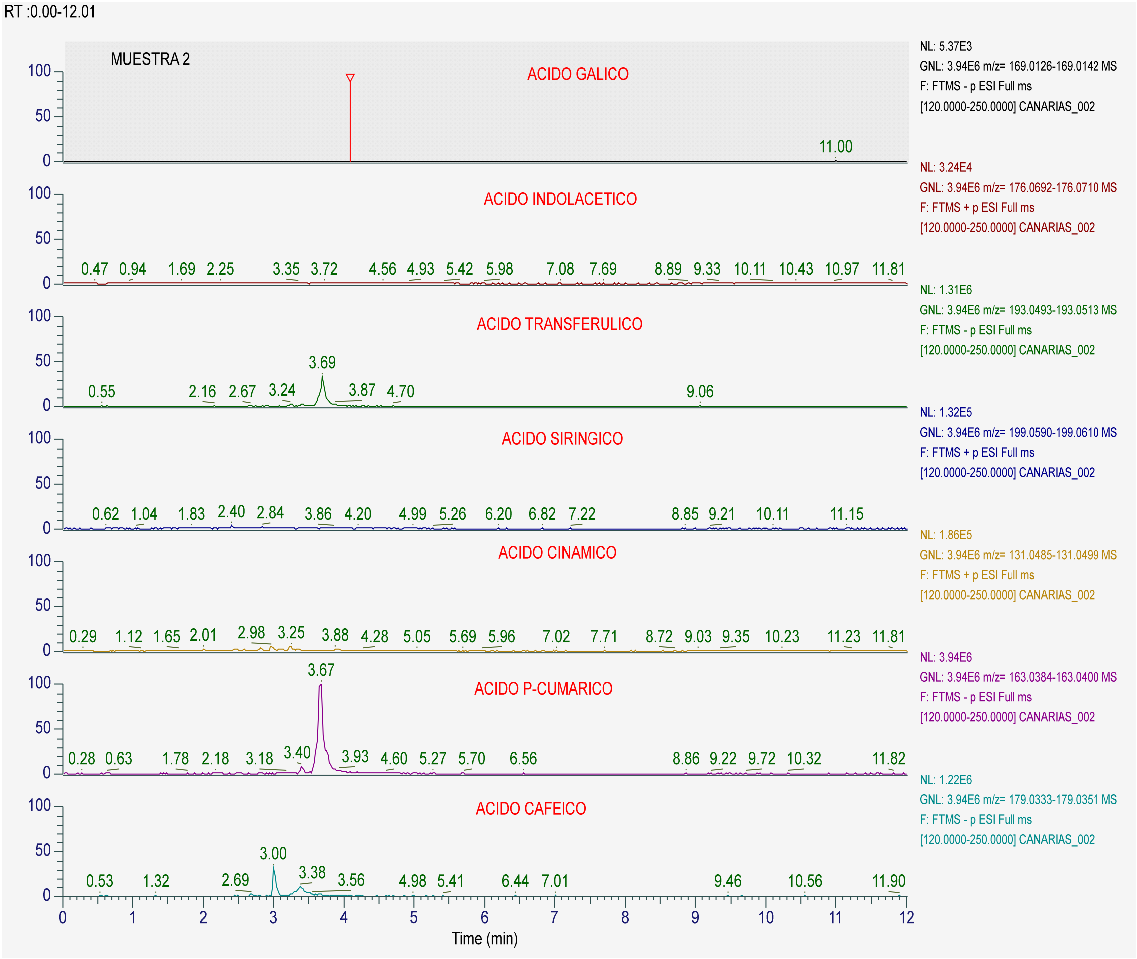

RT :0.00-12.01
MUESTRA 2
ACIDO GALICO
NL: 5.37E3
GNL: 3.94E6 m/z= 169.0126-169.0142 MS
F: FTMS - p ESI Full ms
[120.0000-250.0000] CANARIAS_002
ACIDO INDOLACETICO
NL: 3.24E4
GNL: 3.94E6 m/z= 176.0692-176.0710 MS
F: FTMS + p ESI Full ms
[120.0000-250.0000] CANARIAS_002
ACIDO TRANSFERULICO
NL: 1.31E6
GNL: 3.94E6 m/z= 193.0493-193.0513 MS
F: FTMS - p ESI Full ms
[120.0000-250.0000] CANARIAS_002
ACIDO SIRINGICO
NL: 1.32E5
GNL: 3.94E6 m/z= 199.0590-199.0610 MS
F: FTMS + p ESI Full ms
[120.0000-250.0000] CANARIAS_002
ACIDO CINAMICO
NL: 1.86E5
GNL: 3.94E6 m/z= 131.0485-131.0499 MS
F: FTMS + p ESI Full ms
[120.0000-250.0000] CANARIAS_002
ACIDO P-CUMARICO
NL: 3.94E6
GNL: 3.94E6 m/z= 163.0384-163.0400 MS
F: FTMS - p ESI Full ms
[120.0000-250.0000] CANARIAS_002
ACIDO CAFEICO
NL: 1.22E6
GNL: 3.94E6 m/z= 179.0333-179.0351 MS
F: FTMS - p ESI Full ms
[120.0000-250.0000] CANARIAS_002
Time (min)

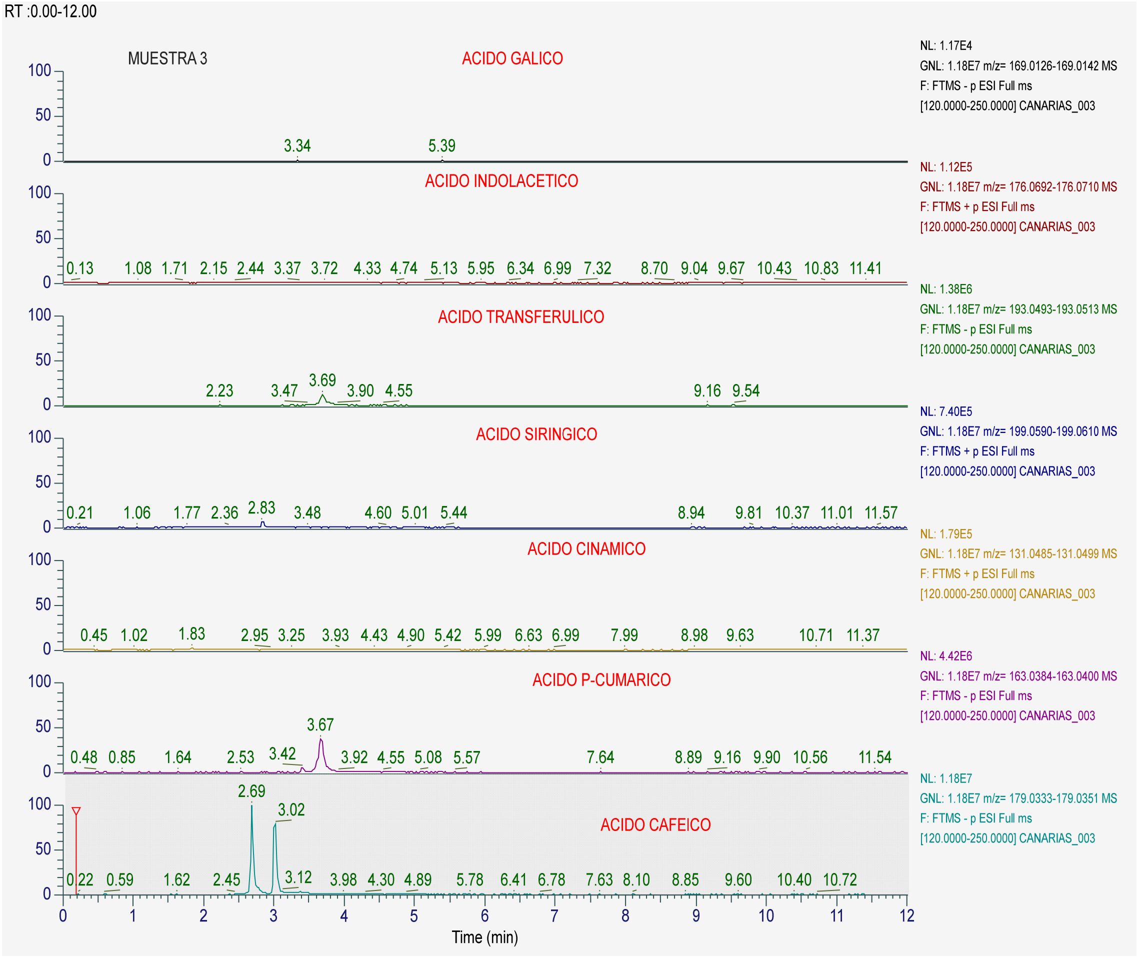
RT :0.00-12.00
MUESTRA 3
ACIDO GALICO
NL: 1.17E4
GNL: 1.18E7 m/z= 169.0126-169.0142 MS
F: FTMS - p ESI Full ms
[120.0000-250.0000] CANARIAS_003
ACIDO INDOLACETICO
NL: 1.12E5
GNL: 1.18E7 m/z= 176.0692-176.0710 MS
F: FTMS + p ESI Full ms
[120.0000-250.0000] CANARIAS_003
ACIDO TRANSFERULICO
NL: 1.38E6
GNL: 1.18E7 m/z= 193.0493-193.0513 MS
F: FTMS - p ESI Full ms
[120.0000-250.0000] CANARIAS_003
ACIDO SIRINGICO
NL: 7.40E5
GNL: 1.18E7 m/z= 199.0590-199.0610 MS
F: FTMS + p ESI Full ms
[120.0000-250.0000] CANARIAS_003
ACIDO CINAMICO
NL: 1.79E5
GNL: 1.18E7 m/z= 131.0485-131.0499 MS
F: FTMS + p ESI Full ms
[120.0000-250.0000] CANARIAS_003
ACIDO P-CUMARICO
NL: 4.42E6
GNL: 1.18E7 m/z= 163.0384-163.0400 MS
F: FTMS - p ESI Full ms
[120.0000-250.0000] CANARIAS_003
ACIDO CAFEICO
NL: 1.18E7
GNL: 1.18E7 m/z= 179.0333-179.0351 MS
F: FTMS - p ESI Full ms
[120.0000-250.0000] CANARIAS_003
Time (min)

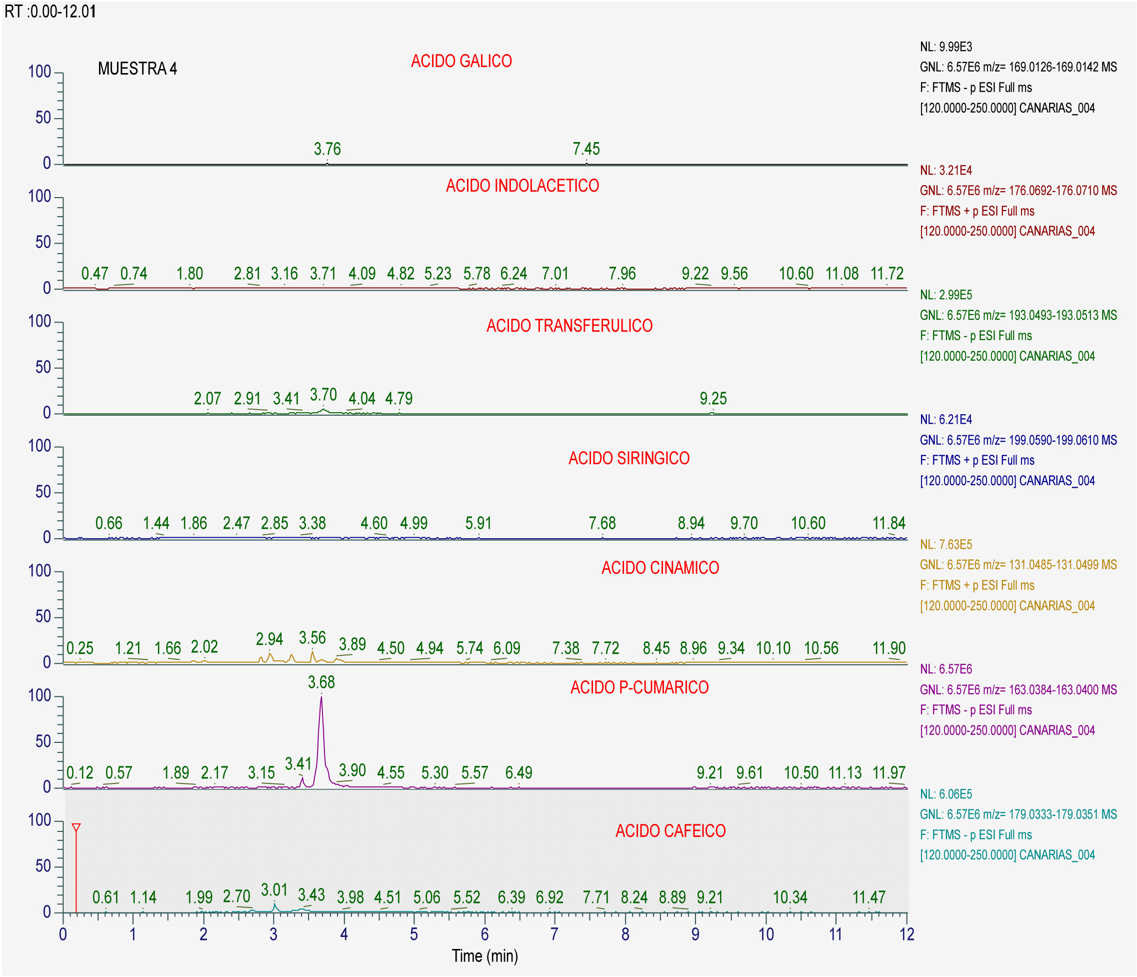

RT :0.00-12.01
MUESTRA 4
ACIDO GALICO
NL: 9.99E3
GNL: 6.57E6 m/z= 169.0126-169.0142 MS
F: FTMS - p ESI Full ms
[120.0000-250.0000] CANARIAS_004
ACIDO INDOLACETICO
NL: 3.21E4
GNL: 6.57E6 m/z= 176.0692-176.0710 MS
F: FTMS + p ESI Full ms
[120.0000-250.0000] CANARIAS_004
ACIDO TRANSFERULICO
NL: 2.99E5
GNL: 6.57E6 m/z= 193.0493-193.0513 MS
F: FTMS - p ESI Full ms
[120.0000-250.0000] CANARIAS_004
ACIDO SIRINGICO
NL: 6.21E4
GNL: 6.57E6 m/z= 199.0590-199.0610 MS
F: FTMS + p ESI Full ms
[120.0000-250.0000] CANARIAS_004
ACIDO CINAMICO
NL: 7.63E5
GNL: 6.57E6 m/z= 131.0485-131.0499 MS
F: FTMS + p ESI Full ms
[120.0000-250.0000] CANARIAS_004
ACIDO P-CUMARICO
NL: 6.57E6
GNL: 6.57E6 m/z= 163.0384-163.0400 MS
F: FTMS - p ESI Full ms
[120.0000-250.0000] CANARIAS_004
ACIDO CAFEICO
NL: 6.06E5
GNL: 6.57E6 m/z= 179.0333-179.0351 MS
F: FTMS - p ESI Full ms
[120.0000-250.0000] CANARIAS_004
Time (min)

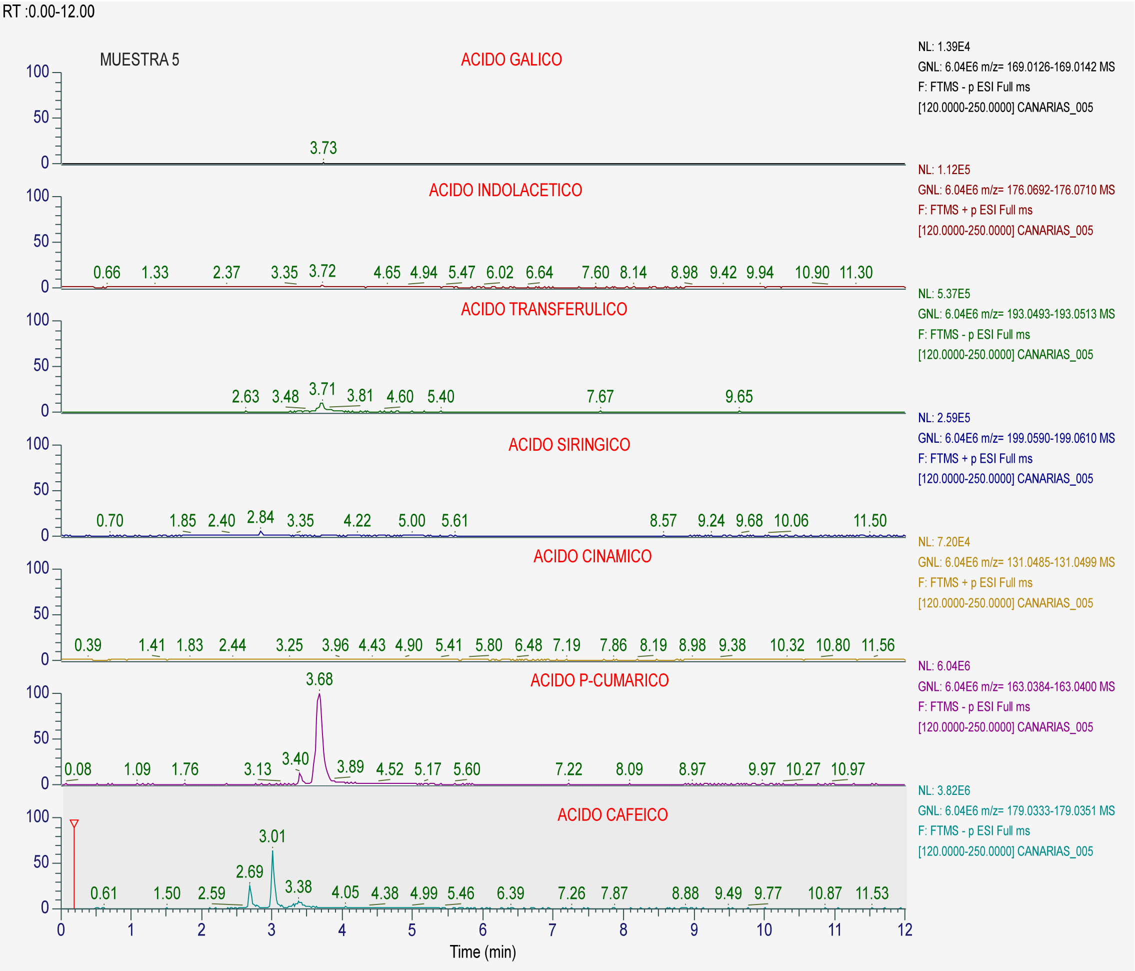
RT :0.00-12.00
MUESTRA 5
ACIDO GALICO
NL: 1.39E4
GNL: 6.04E6 m/z= 169.0126-169.0142 MS
F: FTMS - p ESI Full ms
[120.0000-250.0000] CANARIAS_005
3.73
ACIDO INDOLACETICO
NL: 1.12E5
GNL: 6.04E6 m/z= 176.0692-176.0710 MS
F: FTMS + p ESI Full ms
[120.0000-250.0000] CANARIAS_005
0.66 1.33 2.37 3.35 3.72 4.65 4.94 5.47 6.02 6.64 7.60 8.14 8.98 9.42 9.94 10.90 11.30
ACIDO TRANSFERULICO
NL: 5.37E5
GNL: 6.04E6 m/z= 193.0493-193.0513 MS
F: FTMS - p ESI Full ms
[120.0000-250.0000] CANARIAS_005
2.63 3.48 3.71 3.81 4.60 5.40 7.67 9.65
ACIDO SIRINGICO
NL: 2.59E5
GNL: 6.04E6 m/z= 199.0590-199.0610 MS
F: FTMS + p ESI Full ms
[120.0000-250.0000] CANARIAS_005
0.70 1.85 2.40 2.84 3.35 4.22 5.00 5.61 8.57 9.24 9.68 10.06 11.50
ACIDO CINAMICO
NL: 7.20E4
GNL: 6.04E6 m/z= 131.0485-131.0499 MS
F: FTMS + p ESI Full ms
[120.0000-250.0000] CANARIAS_005
0.39 1.41 1.83 2.44 3.25 3.96 4.43 4.90 5.41 5.80 6.48 7.19 7.86 8.19 8.98 9.38 10.32 10.80 11.56
ACIDO P-CUMARICO
NL: 6.04E6
GNL: 6.04E6 m/z= 163.0384-163.0400 MS
F: FTMS - p ESI Full ms
[120.0000-250.0000] CANARIAS_005
3.68
0.08 1.09 1.76 3.13 3.40 3.89 4.52 5.17 5.60 7.22 8.09 8.97 9.97 10.27 10.97
ACIDO CAFEICO
NL: 3.82E6
GNL: 6.04E6 m/z= 179.0333-179.0351 MS
F: FTMS - p ESI Full ms
[120.0000-250.0000] CANARIAS_005
3.01
2.69
0.61 1.50 2.59 3.38 4.05 4.38 4.99 5.46 6.39 7.26 7.87 8.88 9.49 9.77 10.87 11.53
100
50
0
0 1 2 3 4 5 6 7 8 9 10 11 12
Time (min)

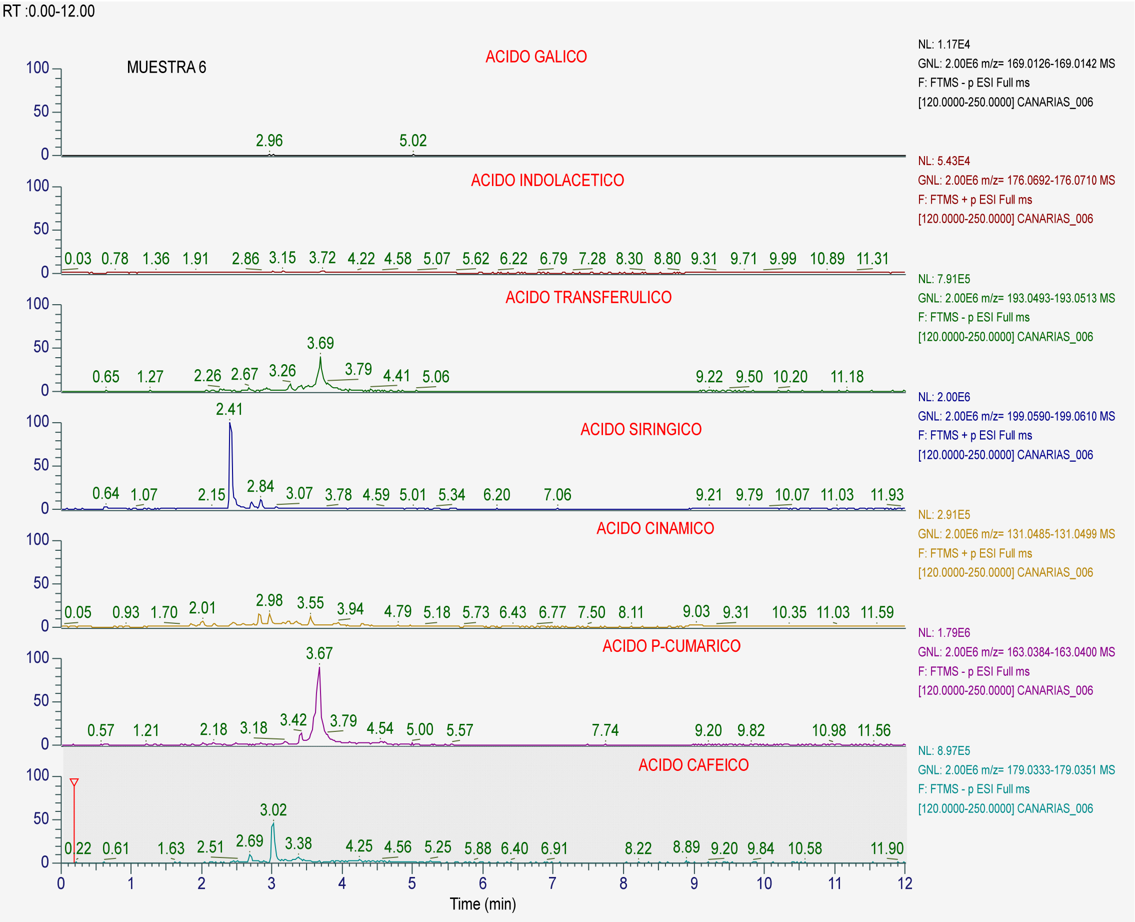
RT :0.00-12.00
MUESTRA 6
ACIDO GALICO
NL: 1.17E4
GNL: 2.00E6 m/z= 169.0126-169.0142 MS
F: FTMS - p ESI Full ms
[120.0000-250.0000] CANARIAS_006
ACIDO INDOLACETICO
NL: 5.43E4
GNL: 2.00E6 m/z= 176.0692-176.0710 MS
F: FTMS + p ESI Full ms
[120.0000-250.0000] CANARIAS_006
ACIDO TRANSFERULICO
NL: 7.91E5
GNL: 2.00E6 m/z= 193.0493-193.0513 MS
F: FTMS - p ESI Full ms
[120.0000-250.0000] CANARIAS_006
ACIDO SIRINGICO
NL: 2.00E6
GNL: 2.00E6 m/z= 199.0590-199.0610 MS
F: FTMS + p ESI Full ms
[120.0000-250.0000] CANARIAS_006
ACIDO CINAMICO
NL: 2.91E5
GNL: 2.00E6 m/z= 131.0485-131.0499 MS
F: FTMS + p ESI Full ms
[120.0000-250.0000] CANARIAS_006
ACIDO P-CUMARICO
NL: 1.79E6
GNL: 2.00E6 m/z= 163.0384-163.0400 MS
F: FTMS - p ESI Full ms
[120.0000-250.0000] CANARIAS_006
ACIDO CAFEICO
NL: 8.97E5
GNL: 2.00E6 m/z= 179.0333-179.0351 MS
F: FTMS - p ESI Full ms
[120.0000-250.0000] CANARIAS_006
Time (min)

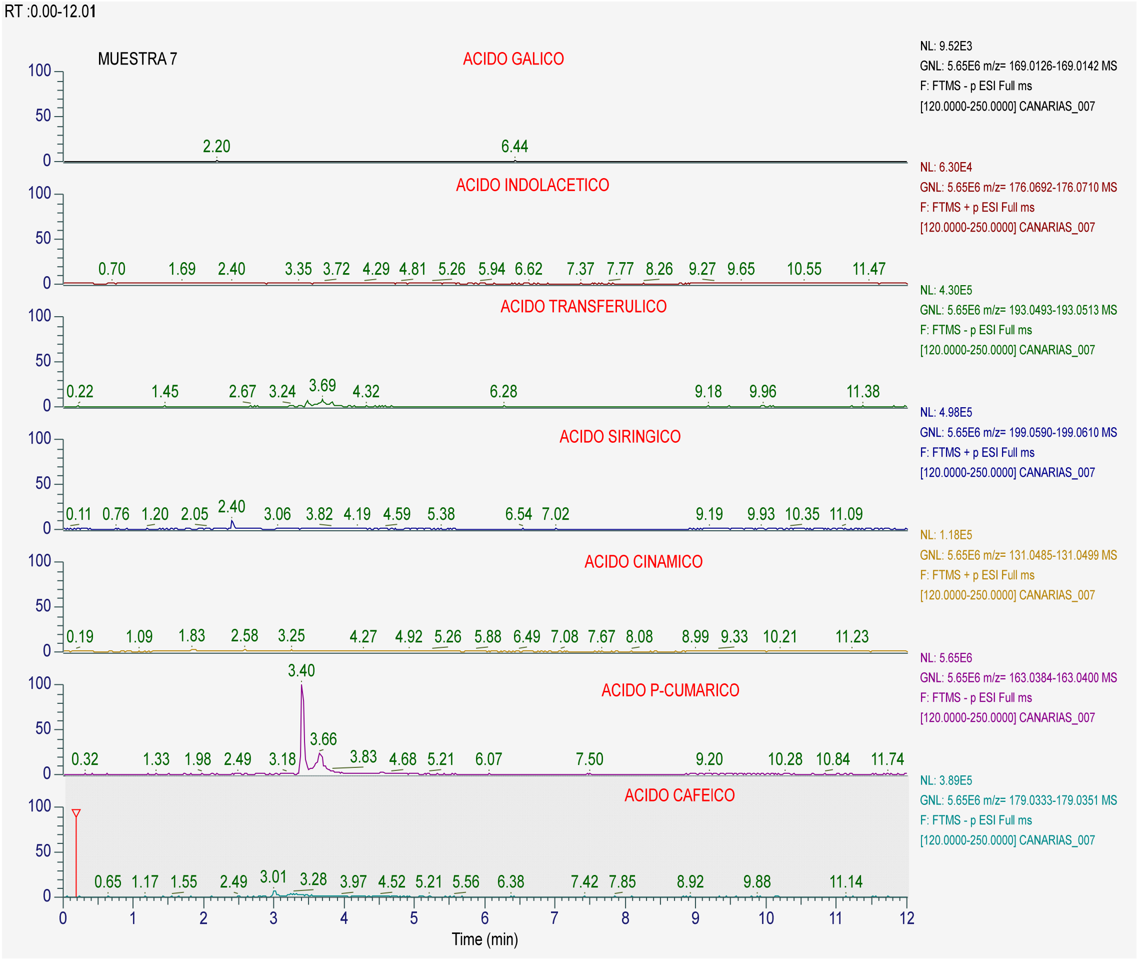
RT :0.00-12.01
MUESTRA 7
ACIDO GALICO
NL: 9.52E3
GNL: 5.65E6 m/z= 169.0126-169.0142 MS
F: FTMS - p ESI Full ms
[120.0000-250.0000] CANARIAS_007
ACIDO INDOLACETICO
NL: 6.30E4
GNL: 5.65E6 m/z= 176.0692-176.0710 MS
F: FTMS + p ESI Full ms
[120.0000-250.0000] CANARIAS_007
ACIDO TRANSFERULICO
NL: 4.30E5
GNL: 5.65E6 m/z= 193.0493-193.0513 MS
F: FTMS - p ESI Full ms
[120.0000-250.0000] CANARIAS_007
ACIDO SIRINGICO
NL: 4.98E5
GNL: 5.65E6 m/z= 199.0590-199.0610 MS
F: FTMS + p ESI Full ms
[120.0000-250.0000] CANARIAS_007
ACIDO CINAMICO
NL: 1.18E5
GNL: 5.65E6 m/z= 131.0485-131.0499 MS
F: FTMS + p ESI Full ms
[120.0000-250.0000] CANARIAS_007
ACIDO P-CUMARICO
NL: 5.65E6
GNL: 5.65E6 m/z= 163.0384-163.0400 MS
F: FTMS - p ESI Full ms
[120.0000-250.0000] CANARIAS_007
ACIDO CAFEICO
NL: 3.89E5
GNL: 5.65E6 m/z= 179.0333-179.0351 MS
F: FTMS - p ESI Full ms
[120.0000-250.0000] CANARIAS_007
Time (min)

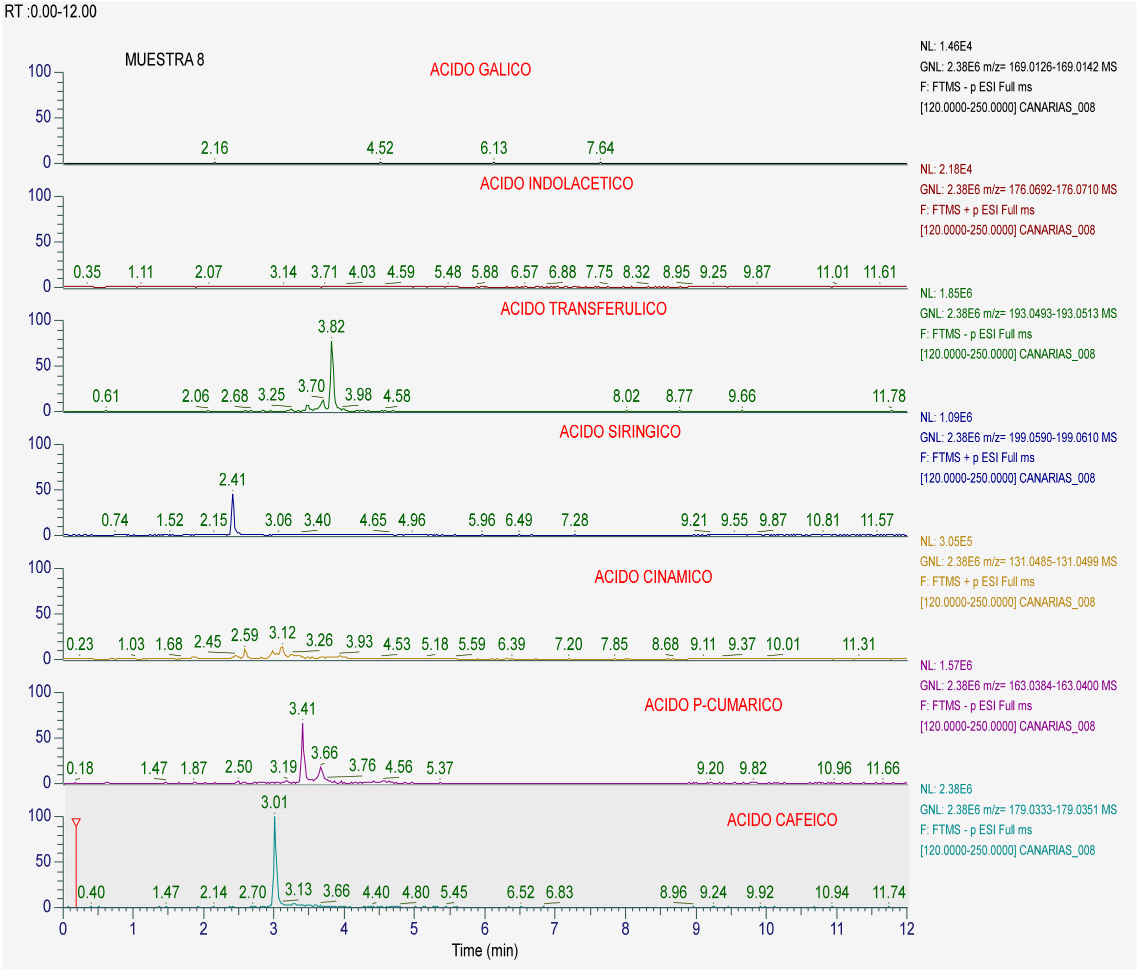

RT :0.00-12.00
MUESTRA 8
ACIDO GALICO
NL: 1.46E4
GNL: 2.38E6 m/z= 169.0126-169.0142 MS
F: FTMS - p ESI Full ms
[120.0000-250.0000] CANARIAS_008
ACIDO INDOLACETICO
NL: 2.18E4
GNL: 2.38E6 m/z= 176.0692-176.0710 MS
F: FTMS + p ESI Full ms
[120.0000-250.0000] CANARIAS_008
ACIDO TRANSFERULICO
NL: 1.85E6
GNL: 2.38E6 m/z= 193.0493-193.0513 MS
F: FTMS - p ESI Full ms
[120.0000-250.0000] CANARIAS_008
ACIDO SIRINGICO
NL: 1.09E6
GNL: 2.38E6 m/z= 199.0590-199.0610 MS
F: FTMS + p ESI Full ms
[120.0000-250.0000] CANARIAS_008
ACIDO CINAMICO
NL: 3.05E5
GNL: 2.38E6 m/z= 131.0485-131.0499 MS
F: FTMS + p ESI Full ms
[120.0000-250.0000] CANARIAS_008
ACIDO P-CUMARICO
NL: 1.57E6
GNL: 2.38E6 m/z= 163.0384-163.0400 MS
F: FTMS - p ESI Full ms
[120.0000-250.0000] CANARIAS_008
ACIDO CAFEICO
NL: 2.38E6
GNL: 2.38E6 m/z= 179.0333-179.0351 MS
F: FTMS - p ESI Full ms
[120.0000-250.0000] CANARIAS_008
Time (min)

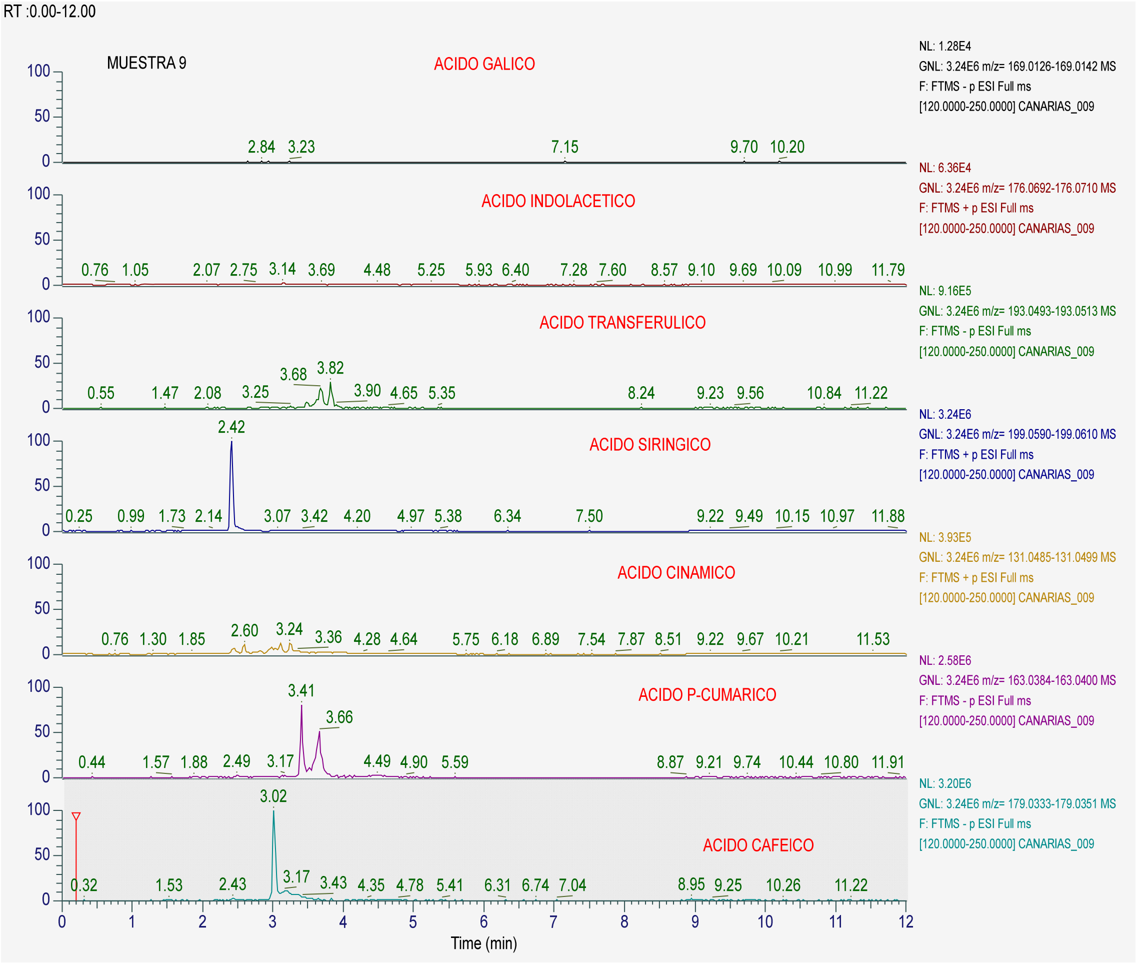
RT :0.00-12.00
MUESTRA 9
ACIDO GALICO
NL: 1.28E4
GNL: 3.24E6 m/z= 169.0126-169.0142 MS
F: FTMS - p ESI Full ms
[120.0000-250.0000] CANARIAS_009
ACIDO INDOLACETICO
NL: 6.36E4
GNL: 3.24E6 m/z= 176.0692-176.0710 MS
F: FTMS + p ESI Full ms
[120.0000-250.0000] CANARIAS_009
ACIDO TRANSFERULICO
NL: 9.16E5
GNL: 3.24E6 m/z= 193.0493-193.0513 MS
F: FTMS - p ESI Full ms
[120.0000-250.0000] CANARIAS_009
ACIDO SIRINGICO
NL: 3.24E6
GNL: 3.24E6 m/z= 199.0590-199.0610 MS
F: FTMS + p ESI Full ms
[120.0000-250.0000] CANARIAS_009
ACIDO CINAMICO
NL: 3.93E5
GNL: 3.24E6 m/z= 131.0485-131.0499 MS
F: FTMS + p ESI Full ms
[120.0000-250.0000] CANARIAS_009
ACIDO P-CUMARICO
NL: 2.58E6
GNL: 3.24E6 m/z= 163.0384-163.0400 MS
F: FTMS - p ESI Full ms
[120.0000-250.0000] CANARIAS_009
ACIDO CAFEICO
NL: 3.20E6
GNL: 3.24E6 m/z= 179.0333-179.0351 MS
F: FTMS - p ESI Full ms
[120.0000-250.0000] CANARIAS_009
Time (min)

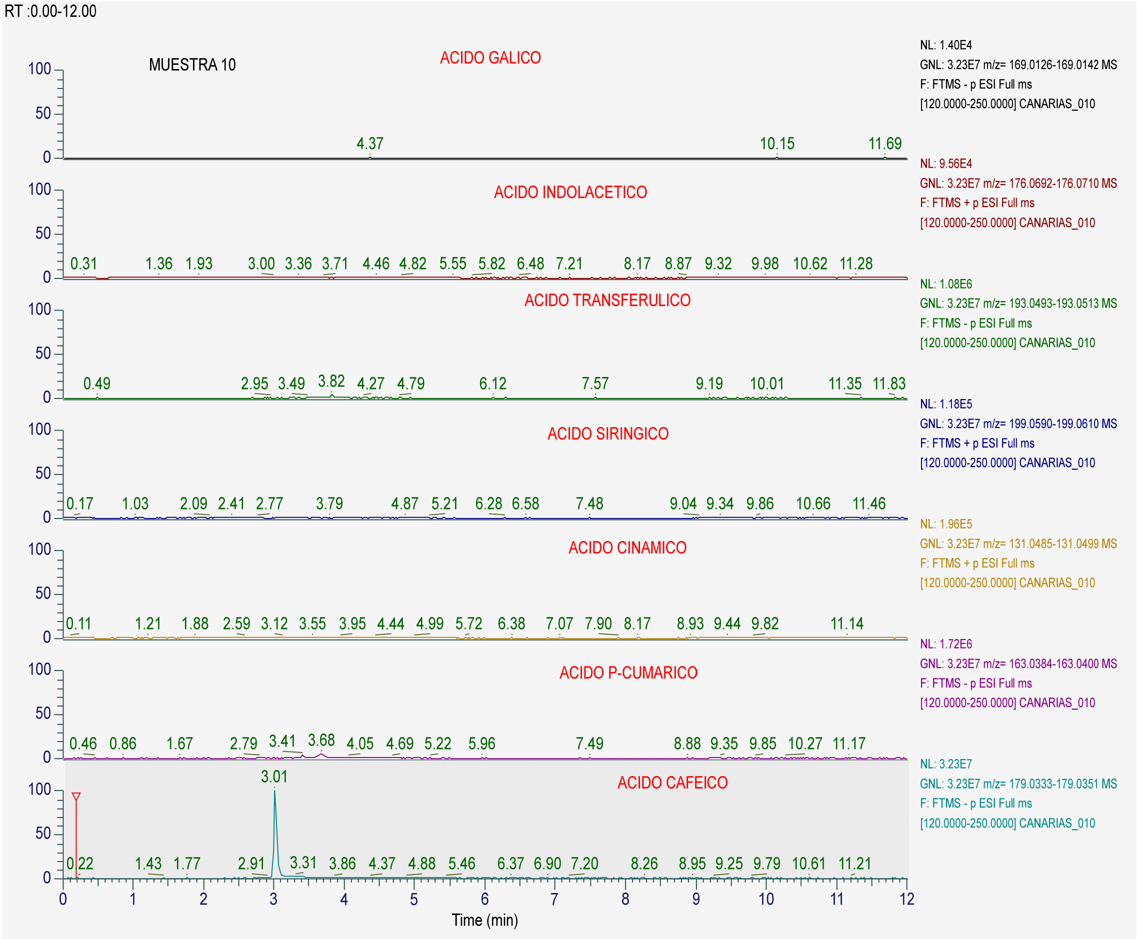
RT :0.00-12.00
MUESTRA 10
ACIDO GALICO
NL: 1.40E4
GNL: 3.23E7 m/z= 169.0126-169.0142 MS
F: FTMS - p ESI Full ms
[120.0000-250.0000] CANARIAS_010
ACIDO INDOLACETICO
NL: 9.56E4
GNL: 3.23E7 m/z= 176.0692-176.0710 MS
F: FTMS + p ESI Full ms
[120.0000-250.0000] CANARIAS_010
ACIDO TRANSFERULICO
NL: 1.08E6
GNL: 3.23E7 m/z= 193.0493-193.0513 MS
F: FTMS - p ESI Full ms
[120.0000-250.0000] CANARIAS_010
ACIDO SIRINGICO
NL: 1.18E5
GNL: 3.23E7 m/z= 199.0590-199.0610 MS
F: FTMS + p ESI Full ms
[120.0000-250.0000] CANARIAS_010
ACIDO CINAMICO
NL: 1.96E5
GNL: 3.23E7 m/z= 131.0485-131.0499 MS
F: FTMS + p ESI Full ms
[120.0000-250.0000] CANARIAS_010
ACIDO P-CUMARICO
NL: 1.72E6
GNL: 3.23E7 m/z= 163.0384-163.0400 MS
F: FTMS - p ESI Full ms
[120.0000-250.0000] CANARIAS_010
ACIDO CAFEICO
NL: 3.23E7
GNL: 3.23E7 m/z= 179.0333-179.0351 MS
F: FTMS - p ESI Full ms
[120.0000-250.0000] CANARIAS_010
Time (min)

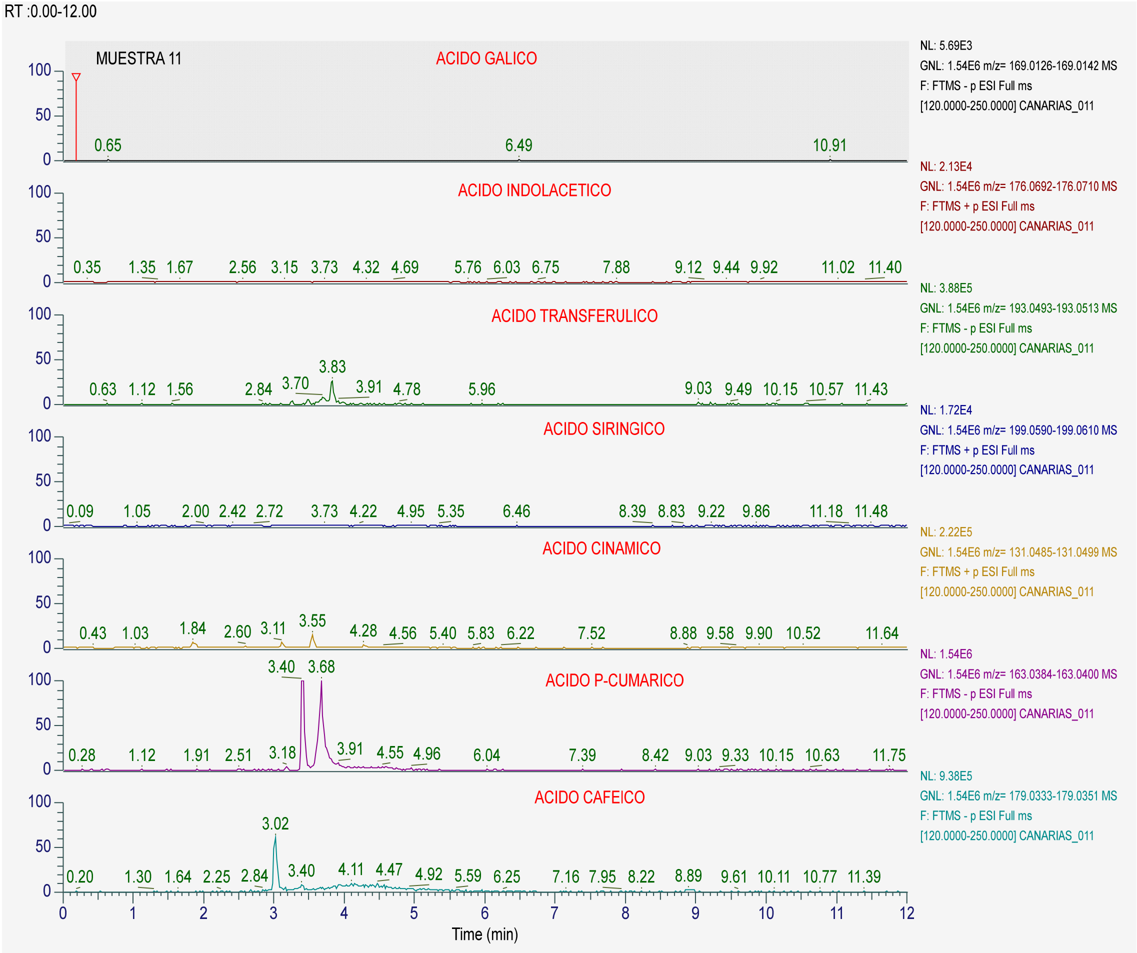
RT :0.00-12.00
MUESTRA 11
ACIDO GALICO
NL: 5.69E3
GNL: 1.54E6 m/z= 169.0126-169.0142 MS
F: FTMS - p ESI Full ms
[120.0000-250.0000] CANARIAS_011
ACIDO INDOLACETICO
NL: 2.13E4
GNL: 1.54E6 m/z= 176.0692-176.0710 MS
F: FTMS + p ESI Full ms
[120.0000-250.0000] CANARIAS_011
ACIDO TRANSFERULICO
NL: 3.88E5
GNL: 1.54E6 m/z= 193.0493-193.0513 MS
F: FTMS - p ESI Full ms
[120.0000-250.0000] CANARIAS_011
ACIDO SIRINGICO
NL: 1.72E4
GNL: 1.54E6 m/z= 199.0590-199.0610 MS
F: FTMS + p ESI Full ms
[120.0000-250.0000] CANARIAS_011
ACIDO CINAMICO
NL: 2.22E5
GNL: 1.54E6 m/z= 131.0485-131.0499 MS
F: FTMS + p ESI Full ms
[120.0000-250.0000] CANARIAS_011
ACIDO P-CUMARICO
NL: 1.54E6
GNL: 1.54E6 m/z= 163.0384-163.0400 MS
F: FTMS - p ESI Full ms
[120.0000-250.0000] CANARIAS_011
ACIDO CAFEICO
NL: 9.38E5
GNL: 1.54E6 m/z= 179.0333-179.0351 MS
F: FTMS - p ESI Full ms
[120.0000-250.0000] CANARIAS_011
Time (min)

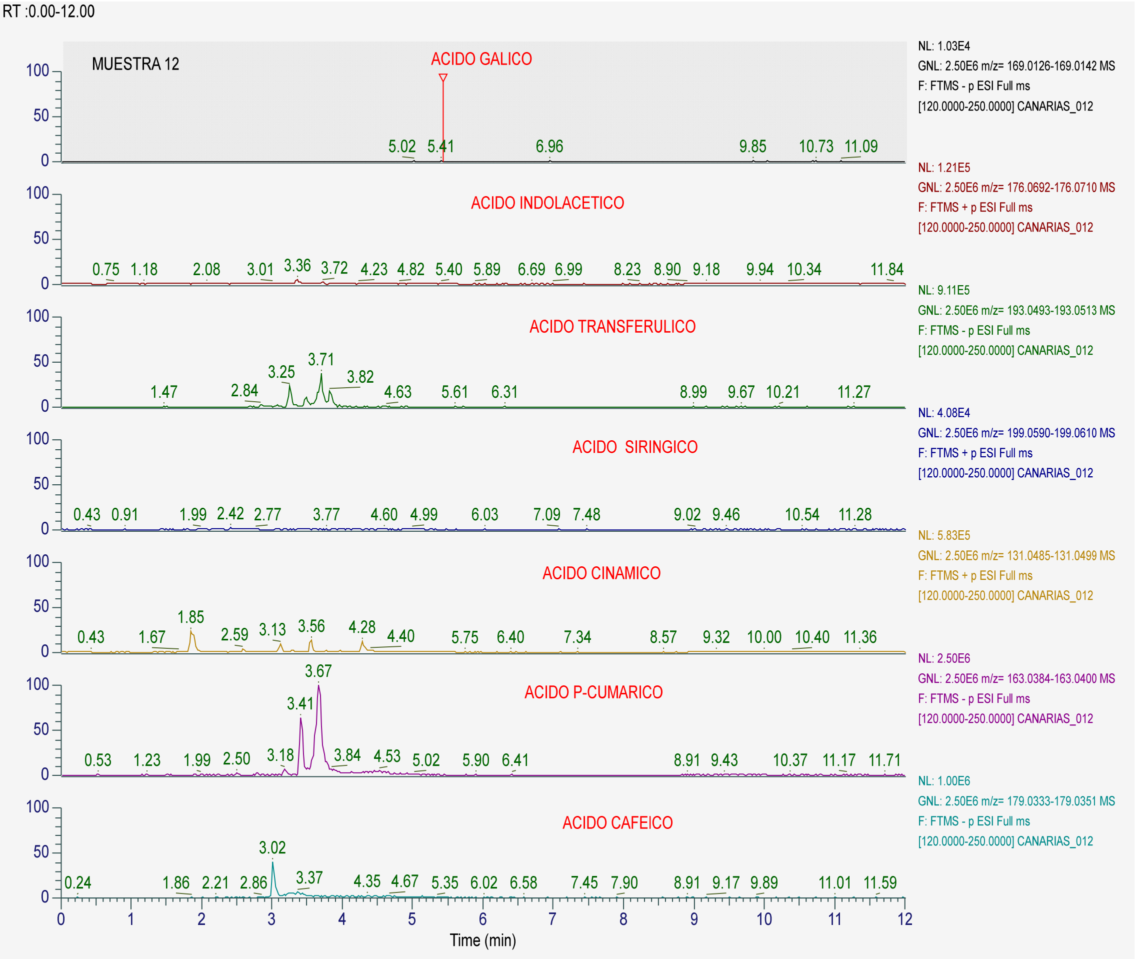

RT :0.00-12.00
MUESTRA 12
ACIDO GALICO
ACIDO INDOLACETICO
ACIDO TRANSFERULICO
ACIDO SIRINGICO
ACIDO CINAMICO
ACIDO P-CUMARICO
ACIDO CAFEICO
Time (min)
NL: 1.03E4
GNL: 2.50E6 m/z= 169.0126-169.0142 MS
F: FTMS - p ESI Full ms
[120.0000-250.0000] CANARIAS_012
NL: 1.21E5
GNL: 2.50E6 m/z= 176.0692-176.0710 MS
F: FTMS + p ESI Full ms
[120.0000-250.0000] CANARIAS_012
NL: 9.11E5
GNL: 2.50E6 m/z= 193.0493-193.0513 MS
F: FTMS - p ESI Full ms
[120.0000-250.0000] CANARIAS_012
NL: 4.08E4
GNL: 2.50E6 m/z= 199.0590-199.0610 MS
F: FTMS + p ESI Full ms
[120.0000-250.0000] CANARIAS_012
NL: 5.83E5
GNL: 2.50E6 m/z= 131.0485-131.0499 MS
F: FTMS + p ESI Full ms
[120.0000-250.0000] CANARIAS_012
NL: 2.50E6
GNL: 2.50E6 m/z= 163.0384-163.0400 MS
F: FTMS - p ESI Full ms
[120.0000-250.0000] CANARIAS_012
NL: 1.00E6
GNL: 2.50E6 m/z= 179.0333-179.0351 MS
F: FTMS - p ESI Full ms
[120.0000-250.0000] CANARIAS_012
